# TDP-43 condensation properties specify its RNA-binding and regulatory repertoire

**DOI:** 10.1016/j.cell.2021.07.018

**Published:** 2021-09-02

**Authors:** Martina Hallegger, Anob M. Chakrabarti, Flora C.Y. Lee, Bo Lim Lee, Aram G. Amalietti, Hana M. Odeh, Katie E. Copley, Jack D. Rubien, Bede Portz, Klara Kuret, Ina Huppertz, Frédérique Rau, Rickie Patani, Nicolas L. Fawzi, James Shorter, Nicholas M. Luscombe, Jernej Ule

**Affiliations:** 1 The Francis Crick Institute, 1 Midland Road, London NW1 1AT, UK; 2 Department of Neuromuscular Diseases, UCL Queen Square Institute of Neurology, Queen Square, London WC1N 3BG, UK; 3 Department of Genetics, Evolution and Environment, UCL Genetics Institute, Gower Street, London WC1E 6BT, UK; 4 Department of Biochemistry and Biophysics, Perelman School of Medicine at the University of Pennsylvania, Philadelphia, PA 19104, USA; 5 National Institute of Chemistry, Hajdrihova 19, 1001 Ljubljana, Slovenia; 6 Neuroscience Graduate Group, Perelman School of Medicine at the University of Pennsylvania, Philadelphia, PA 19104, USA; 7 European Molecular Biology Laboratory, Meyerhofstrasse 1, 69117 Heidelberg, Germany; 8 Department of Molecular Pharmacology, Physiology, and Biotechnology, Brown University, Providence, RI 02912, USA; 9 Okinawa Institute of Science & Technology Graduate University, 1919-1 Tancha, Onna-son, Kunigami-gun, Okinawa 904-0495, Japan

**Keywords:** RNA-binding protein, TDP-43, iCLIP, condensation, RNA granules, phase separation, intrinsically disordered region, amyotrophic lateral sclerosis, multivalency, alternative polyadenylation

## Abstract

Mutations causing amyotrophic lateral sclerosis (ALS) often affect the condensation properties of RNA-binding proteins (RBPs). However, the role of RBP condensation in the specificity and function of protein-RNA complexes remains unclear. We created a series of TDP-43 C-terminal domain (CTD) variants that exhibited a gradient of low to high condensation propensity, as observed *in vitro* and by nuclear mobility and foci formation. Notably, a capacity for condensation was required for efficient TDP-43 assembly on subsets of RNA-binding regions, which contain unusually long clusters of motifs of characteristic types and density. These “binding-region condensates” are promoted by homomeric CTD-driven interactions and required for efficient regulation of a subset of bound transcripts, including autoregulation of *TDP-43* mRNA. We establish that RBP condensation can occur in a binding-region-specific manner to selectively modulate transcriptome-wide RNA regulation, which has implications for remodeling RNA networks in the context of signaling, disease, and evolution.

## Highlights

• TDP-43 mutants affect condensation properties to a similar extent at multiple scales
• Binding-region condensates form on long RNA regions with dispersed UG-rich motifs
• RBPchimera-CLIP indicates homomeric interactions promote molecular-scale condensates
• Condensation selectively tunes the regulatory capacity of TDP-43; e.g., autoregulation

## Graphical abstract

**Figure:**
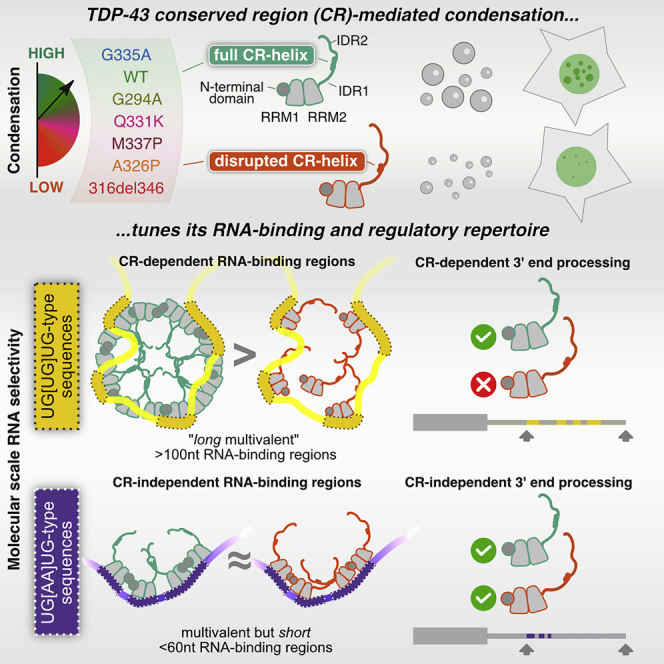

## Teaser

The condensation propensity of an RNA-binding protein tunes its binding to specific RNA regions across the transcriptome and affects its RNA processing functions. Formation of these “binding-region condensates,” promoted by specific motif types that are dispersed across long RNA regions, expands the ways in which RNA binding can be selectively controlled beyond canonical RNA-binding domains.

## Introduction

Changes in the activity of RNA-binding proteins (RBPs) play crucial roles in shaping cell-type-specific gene regulation and signal responses. Many mechanisms modulate the activity of an RBP, such as changes in abundance, localization, or condensation of ribonucleoprotein complexes (RNPs) (Alberti and Hyman, 2021). Condensation has been studied by monitoring liquid-liquid phase separation (LLPS) of purified RBPs and RNAs or formation of phase-separated RNA granules in cells, but it can also occur at molecular nanometer scales (Lyon et al., 2021). RBP condensation is often mediated by intrinsically disordered regions (IDRs) rich in small, polar, and charged amino acids, which are capable of weak multivalent interactions. Condensation properties are commonly modified by mutations or post-translational modifications of IDRs (Chong and Forman-Kay, 2016; Lyon et al., 2021). Indeed, over a dozen RBP-coding genes are associated with amyotrophic lateral sclerosis (ALS), and many of the causal mutations modify the RBPs’ condensation properties (Harrison and Shorter, 2017; Kim et al., 2013; Patel et al., 2015). However, it is unknown whether changes in RBP condensation selectively affect binding and regulation of specific RNAs, and if so, which RNA features might mediate such selectivity.

**Figure 1:**
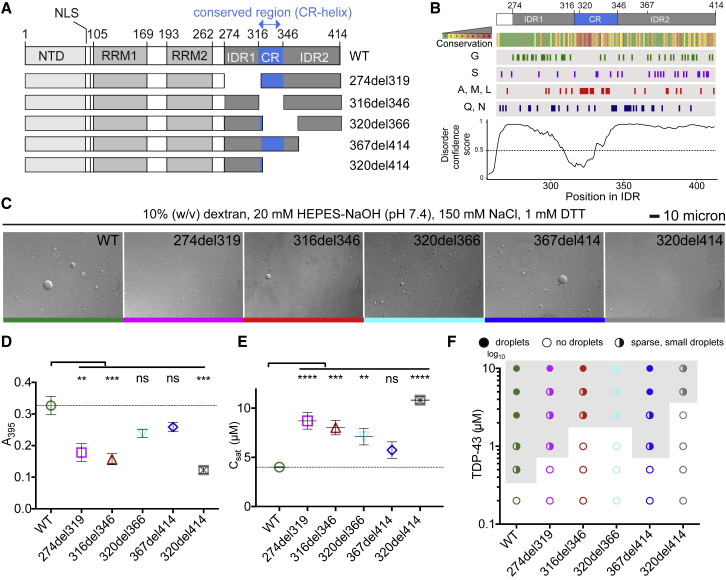
Deletions within the CR change the condensation behavior of TDP-43 *in vitro* (A) The domain map of TDP-43 includes an N-terminal domain (NTD), two RNA recognition motifs (RRM1 and RRM2), two intrinsically disordered regions (IDR1, 2) at the C terminus with an intervening conserved region (CR helix), and a nuclear localization signal (NLS; residues 82–92). The positions of the 5 deletion variants are shown. (B) Disorder confidence score, amino acid conservation (green, least conserved; red, most conserved position), and compositional biases of the C-terminal domain (STAR Methods). (C) Differential interference contrast (DIC) microscopy images of 10 μM WT MBP-TDP-43 and deletion variants show differences in droplet formation. The scale bar represents 10 μm. (D) Turbidity measurements of phase-separated WT MBP-TDP-43 and deletion variants. Mean (±SEM), n = 3, one-way ANOVA (^∗∗^p < 0.01 and ^∗∗∗^p < 0.001), significant against the WT. The dashed line indicates the WT absorbance value. (E) C_sat_ (μM) of TDP-43 deletion constructs were determined by measuring the supernatant concentration after LLPS at 10 μM. Mean (±SEM), n = 3, one-way ANOVA shows significant difference against WT TDP-43 (^∗∗∗^p < 0.001). The dashed line indicates the C_sat_ of the WT. (F) Phase diagram showing changes in the phase boundary of deletion variants. “Sparse, small droplets” refer to droplets as in 320del414 in (C). The experiment was repeated three times.

A central RBP in ALS pathogenesis is TDP-43 (*trans*-activating response element DNA-binding protein of 43 kDa). Post-mortem tissue from ∼97% of individuals with ALS presents TDP-43 aggregates, and TDP-43 proteinopathy is also common in frontotemporal dementia (FTD), limbic-predominant age-related TDP-43 encephalopathy (LATE) and Alzheimer’s disease (Gao et al., 2019; Nelson et al., 2019). Moreover, mutations in *TARDBP*, the gene encoding TDP-43, can cause ALS (Chia et al., 2018; Sreedharan et al., 2008). Most mutations modify the C-terminal domain (CTD) of TDP-43, which comprises two disordered regions (IDR1 and IDR2) and a short conserved region (CR); all three contain sites that can form weak homomeric contacts that promote TDP-43 condensation (Tziortzouda et al., 2021; Figures 1A and 1B). At a high local concentration of TDP-43, the CR adapts an α-helix fold (CR helix) that is stabilized by homomeric contacts between CR helices of adjacent TDP-43 molecules (Conicella et al., 2016). ALS-causing mutations in the CR can affect helix formation and the capacity of a CR-helix for homomeric contacts (Conicella et al., 2016, 2020). For the purpose of this study, any phenomena that require an intact CR helix are considered to require TDP-43 condensation, at least at a molecular scale.

In this study, we examined whether TDP-43 condensation contributes to its RNA binding and regulation and whether it involves entire RNAs or regions of RNAs. We found that point mutations in the CR have the same gradient of effects at multiple scales: *in vitro* LLPS, formation and dynamics of TDP-43 foci in cell nuclei, binding to specific RNA regions, and selective regulation of RNA processing. Given that condensation selectively contributes to only some of the bound RNA regions, we wanted to find out which RNA features mediate such selectivity. Multivalency, clustering of multiple binding motifs, is a feature of RNAs that can promote condensation of bound RBPs (Li et al., 2012; Lyon et al., 2021). However, binding regions of TDP-43 are generally highly multivalent, and the sensitivity to CR helix mutations additionally depends on a dispersed arrangement of specific motif types over long multivalent regions (generally more than 100 nt). We show that altered condensation properties of TDP-43 selectively modify its RNA-regulatory network.

## Results

### The CR is essential for efficient LLPS of full-length TDP-43

Several regions of TDP-43 are capable of higher-order interactions. The CTD is sufficient for LLPS, but the specific roles of each region within the CTD are unclear (Tziortzouda et al., 2021). To identify critical regions, we produced TDP-43 deletion constructs omitting sections of the CTD (Figures 1A and 1B): 274del319 removes IDR1, which is rich in glycines; 316del346 removes the helix-forming CR, which is rich in alanine, methionine, and leucine (Figure 1B); 320del366 removes most of the CR along with a glutamine- and asparagine-rich region that resembles yeast prion domains (Alberti et al., 2009); 367del414 removes the glycine- and serine-rich IDR2; and 320del414 removes most of the CR and all of IDR2. Under *in vitro* conditions, with dextran as a crowding agent, 10 μM of purified full-length wild-type (WT) TDP-43 (with a C-terminal maltose-binding protein (MBP) tag) spontaneously formed droplets (Wang et al., 2018). These conditions mimic the 1–10 μM physiological concentration of TDP-43 (Ling et al., 2010). All mutants affected droplet formation to variable extents (Figures 1C–1F), indicating that each deleted region contributes to TDP-43 condensation.

**Figure S1:**
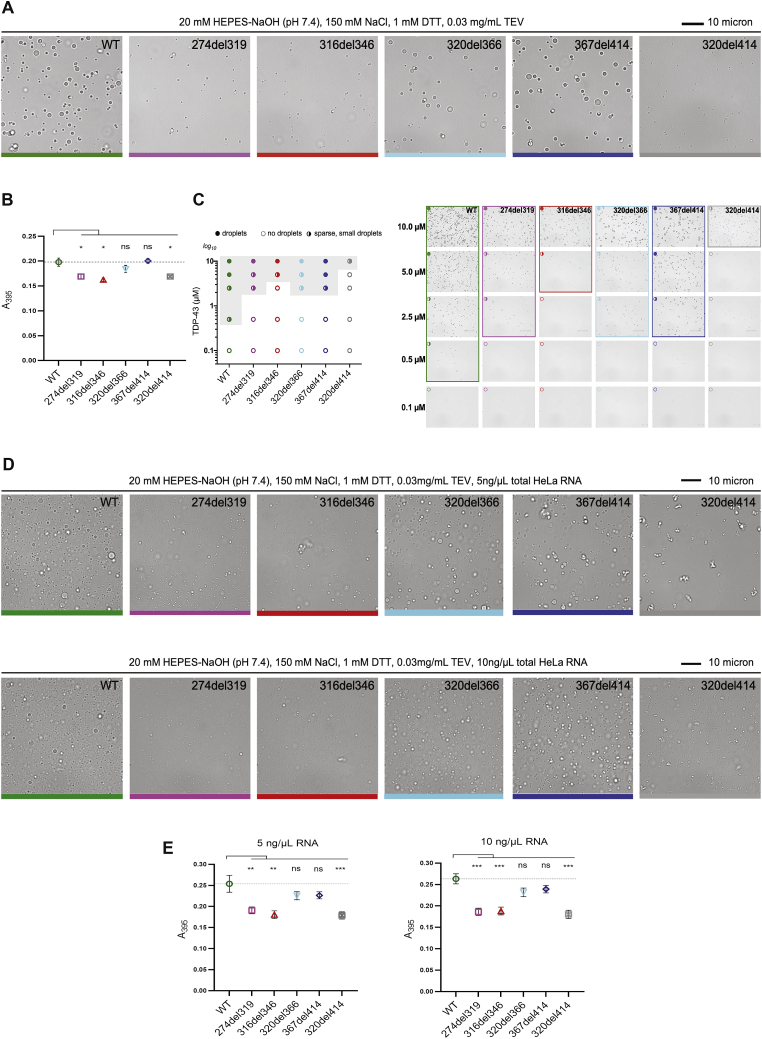
Deletions within the CR change the condensation behavior of TDP-43, related to Figure 1 A. Brightfield microscopy images of WT TDP-43-MBP and deletion variants (5μM) after addition of TEV protease show differences in droplet formation. The black bar represents 10 μm. B. Turbidity measurements of phase-separated WT TDP-43-MBP and deletion variants (5μM) after addition of TEV protease. Mean (±SEM), n = 2, one-way ANOVA (^∗^p < 0.05). Asterisks, significant relative to the WT. The dashed line indicates the absorbance value of WT. C. Phase diagram (left) shows changes in the phase boundary of deletion variants. Representative brightfield microscopy images are shown on the right. Scale bar, 10μm. The experiment was repeated two times. D. Brightfield microscopy images of WT TDP-43-MBP and deletion variants (10μM) after addition of TEV protease in the presence of 5ng/μl (upper panels) or 10ng/μl (lower panels) of total HeLa cell RNA show differences in droplet formation. The black bar represents 10 μm. E. Turbidity measurements of phase-separated WT TDP-43-MBP and deletion variants (10μM) after addition of TEV protease in the presence of 5ng/μl (left) or 10ng/μl (right) of total HeLa cell RNA. Mean (±SEM), n = 3-4, one-way ANOVA (^∗∗^p < 0.005; ^∗∗∗^p < 0.001). Asterisks, significant relative to the WT. The dashed line indicates the absorbance value of WT.

The saturation concentration (C_sat_) for each mutant was determined after separation of droplets by centrifugation. All mutants showed increased C_sat_ (Figure 1E) and a shift in the phase boundary (gray area in Figure 1F), demonstrating that higher protein concentrations are required for LLPS. The smallest perturbation was seen for 367del414, the only deletion that does not disrupt the CR, whereas the 316del346 deletion, which removes only the CR, strongly disrupted LLPS (Figures 1C–1F). These results were not due to the presence of the MBP tag or crowding agent because TDP-43 spontaneously formed droplets in the absence of dextran when the MBP tag was cleaved from TDP-43 with tobacco etch virus (TEV) protease (Figures S1A–S1C; Wang et al., 2018). Moreover, the varying propensities for LLPS were unaffected by addition of total HeLa cell RNA at 5 ng/μL or 10 ng/μL (Figures S1D and S1E), whereas higher concentrations of total HeLa cell RNA (40 ng/μL) inhibited TDP-43 LLPS (data not shown), as anticipated from similar findings with yeast total RNA (Mann et al., 2019). Our results demonstrate that the CR plays a central role in LLPS of full-length TDP-43, in agreement with its requirement for LLPS of the truncated protein (Conicella et al., 2016).

### CR promotes condensation of TDP-43 in cell nuclei

**Figure S2:**
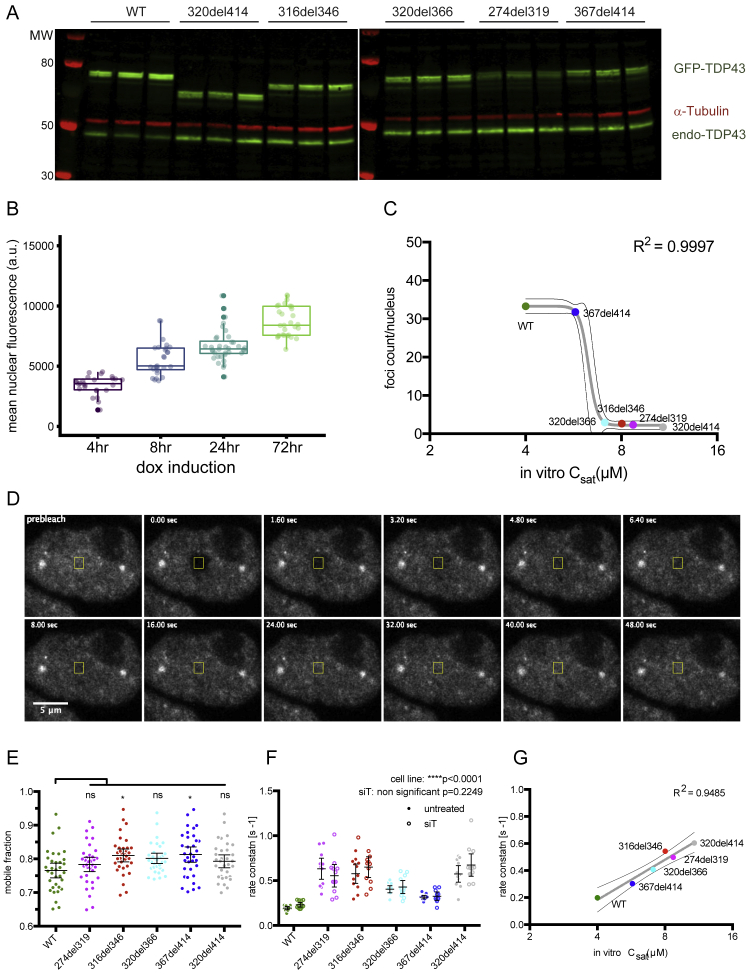
Deletions within the CR change the condensation behavior of TDP-43 in cells, related to Figure 2 A. Western blot analysis of expression level of the various dox-inducible constructs, compared to the endogenous TDP-43, as determined by the anti-TDP-43 antibody. B. Quantification of mean nuclear fluorescence levels of GFP-TDP-43 WT Flp-In cells induced with dox for 4, 8, 24 and 72hrs. n (cells) = 26, 27, 36, 29. C. Relationship between *in vitro* C_sat_ (μM) of purified TDP-43 deletion constructs (Figure 1E) and quantification of foci counts per nucleus in confocal images of HEK293 Flp-In cell lines expressing the dox-inducible GFP-TDP-43 variants (Figure 2E). The sigmoidal curve (±95% confidence band) shown was fitted to the means of each datapoint. D. Representative image series and ROI from nucleoplasm FRAP experiments (Figure 2F) of GFP-TDP-43 WT cells. E. Mobile fraction of TDP-43, obtained from the plateau of the fitted exponential curve from FRAP data shown in Figure 2F. Mean ± 95%CI are shown for n (cells) = 34 for all cell lines. Significance was tested with Kruskal-Wallis test followed by Dunn’s Multiple Comparison Test. The p values reported are for the individual comparisons (^∗^p adj. < 0.05). F. Rate constant of FRAP from WT and deletion constructs with or without siRNA mediated knockdown of endogenous TDP-43 (siT). Mean ± 95%CI are shown for n (cells) = 12 for all conditions except n (cells) = 11 for 367del414 siT. Significance was tested with Two-way ANOVA (cell line: ^∗∗∗∗^p < 0.0001, siT: not significant p = 0.2249, interaction: not significant p = 0.3052). Rate constant was not found to be significantly different between all pairs of untreated versus si-TDP-43 conditions. G. Relationship between *in vitro* C_sat_ (μM) of purified TDP-43 deletion constructs (Figure 1E) and FRAP rate constants assessing mobility of GFP-TDP-43 in HEK293 Flp-In cell lines (Figure 2G). The linear regression (±95% confidence band) shown was fitted to the means of each datapoint.

**Figure 2:**
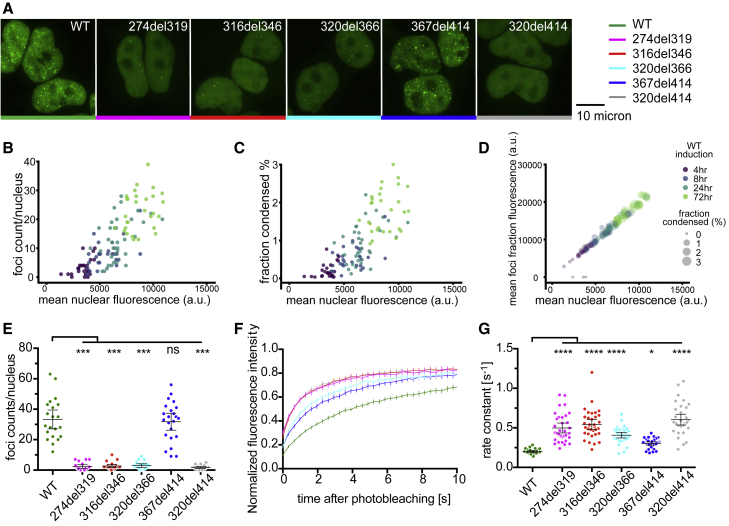
Deletions within the CR change the condensation behavior of TDP-43 in cells (A) Maximum z-projection images of HEK293 Flp-In cell lines expressing doxycycline (dox)-inducible GFP-TDP-43 variants. (B) Heterogeneous expression levels of GFP-TDP-43 WT were induced with a dox time course (4–72 h) in HEK293 Flp-In cells, n (cells) = 118. Foci counts in each segmented nucleus are plotted against the total nuclear fluorescence of individual GFP-TDP-43 WT cells. (C) As in (B), the fractional nuclear area occupied by the sum of all foci in each nucleus plotted against the mean nuclear fluorescence. (D) As in (B), the relationship between the mean foci fraction fluorescence intensity and mean nuclear fluorescence. (E) Quantification of foci counts in each segmented nucleus. Mean ± 95% confidence interval (CI) is shown. n (cells): WT = 21, 274del319 = 20, 316del346 = 22, 320del366 = 20, 367del414 = 23, 320del414 = 23. (F) FRAP experiments on HEK293 cell lines. The fluorescence recovery curve was obtained by bleaching a spot of predefined size in the nucleoplasm. Mean ± 95% CI is shown for 34 cells for all cell lines. (G) Rate constant of fluorescence recovery. Mean ± 95% CI is shown. n (cells) = 34 for all cell lines. Significance for (E) and (G) was tested with Kruskal-Wallis test followed by Dunn’s multiple comparisons test. Reported adjusted (adj.) p values are for the individual comparisons (^∗^p adj. < 0.05, ^∗∗∗^p adj. < 0.001, and ^∗∗∗∗^p adj. < 0.0001).

RBP condensation can lead to formation of microscopically visible granules (Lyon et al., 2021). To address the role of IDRs in RBP condensation under physiological conditions, we generated stable Flp-In HEK293 cell lines with doxycycline-inducible expression of small interfering RNA (siRNA)-resistant N-terminally GFP-tagged WT or mutant GFP-TDP-43. 24 h after induction, GFP-TDP-43 was expressed at similar levels as endogenous TDP-43 (Figure S2A). WT and mutant versions of GFP-TDP-43 were predominantly localized in the nucleus, with diffuse signals present across the nucleoplasm and additional punctate patterns we refer to as “nuclear foci” (Figure 2A). To determine the number and area of these foci, we automated the nuclear segmentation and foci-counting procedure on confocal z stack images. An induction time course of 4–72 h showed that *in vivo* assembly of TDP-43 foci involves a concentration-dependent condensation process (Figures 2B–2D and S2B). At 24 h, there is a substantially reduced number of foci in all mutant GFP-TDP-43 lines, except for 367del414, in which the CR is preserved (Figure 2E). The extent of nuclear foci formation in WT and deletion cell lines followed a sigmoidal relationship with C_sat_ measurements of the purified proteins (R^2^ = 0.9997; Figure S2C), indicating that the threshold for foci formation in the nucleus is deletion dependent. Thus, TDP-43 nuclear foci formation and its concentration dependence is likely partially modified by the weak CR helix-mediated homomeric interactions that mirror LLPS behavior under *in vitro* conditions.

To further investigate the effect of CTD deletions on TDP-43 mobility in cells, we employed fluorescence recovery after photobleaching (FRAP) of GFP-TDP-43. We photobleached regions of the nucleoplasm with similar GFP intensities and monitored the rate of signal recovery (Figure S2D). GFP-TDP-43 mobility was increased in all mutant lines except 367del414, which showed only a small increase. The effect was independent of protein size, with small deletions having a similar effect as the large 320del414 deletion (Figures 2F and 2G). The proportions of protein in the mobile fraction calculated from FRAP experiments are very similar for all constructs during the observed time period (Figure S2E). We examined the mobility of GFP-TDP-43 in cells with or without siRNA-mediated depletion of endogenous TDP-43 and observed no statistically significant change between the two treatments for the WT or any mutant (Figure S2F). Thus, the presence of endogenous TDP-43 has no effect on the mobility of any GFP-TDP-43 construct. Again, the extent of increased mobility in cell nuclei, as measured by FRAP, correlated with the degree of perturbation of *in vitro* LLPS for the different deletions (R^2^ = 0.9485; Figure S2G). We find that CR-disrupting deletions had strong correlated effects on LLPS, the number of nuclear foci, and the mobility of GFP-TDP-43. This indicates that these distinct measurements likely detect shared aspects of condensation propensity and demonstrate that the CR is essential for optimal TDP-43 condensation *in vitro* and in cell nuclei.

### CR point mutants of TDP-43 have a gradient of condensation properties

**Figure 3:**
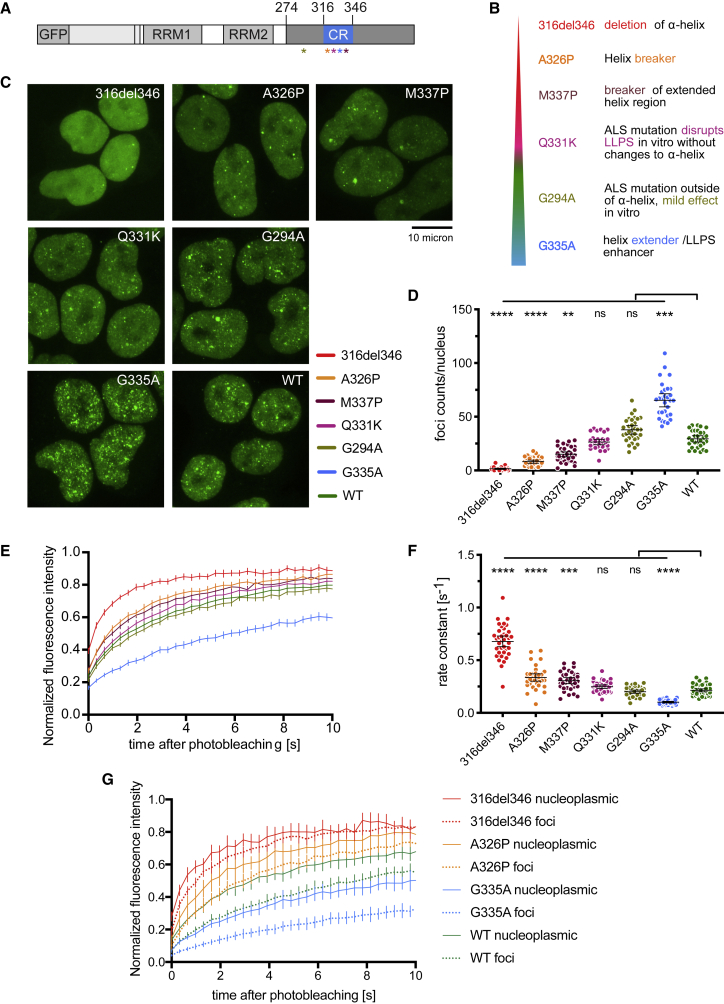
CR point mutants of TDP-43 have a gradient of *in vivo* condensation properties (A) Positions of the 5 point mutations in TDP-43 variants. (B) Mutations modify condensation on a gradient from perturbing (red/orange) to maintaining (green) or promoting (blue) the condensation capacity. (C) Maximum z-projection images of HEK293 Flp-In cell lines expressing the indicated dox-inducible GFP-TDP-43 variants. (D) Quantification of foci counts in each segmented nucleus of confocal z stacks. Mean ± 95% CI is shown for n (cells): 316del346 = 30, A326P = 32, M337P = 35, Q331K = 32, G294A = 32, G335A = 30, WT = 30. (E) FRAP experiments on GFP-TDP-43 CR mutant cell lines. The fluorescence recovery curve was obtained after bleaching a spot of predefined size in the nucleoplasm. Mean ± 95% CI is shown for n (cells): 316del346 = 37, A326P = 36, M337P = 36, Q331K = 36, G294A = 36, G335A = 36, WT = 48. (F) Rate constant of fluorescence recovery. Mean ± 95% CI is shown for the same number of cells as in (E). Significance for (D) and (F) was tested with Kruskal-Wallis test followed by Dunn’s multiple comparisons test. Reported p values are for the individual comparisons (^∗∗^p adj. < 0.01, ^∗∗∗^p < 0.001, and ^∗∗∗∗^p < 0.0001). (G) As in (E) for FRAP analysis of GFP TDP-43 mobility in nucleoplasm versus nuclear regions centered on foci on 316del346, A326P, G335A, and WT GFP-TDP-43 cell lines. Mean ± 95% CI is shown for n (cells): 316del346 nucleoplasm = 8, 316del346 foci = 12, A326P nucleoplasm = 8, A326P foci = 12, G335A nucleoplasm = 8, G335A foci = 12, WT nucleoplasm = 8, WT foci = 10, 1 focus per independent cell.

**Figure S3:**
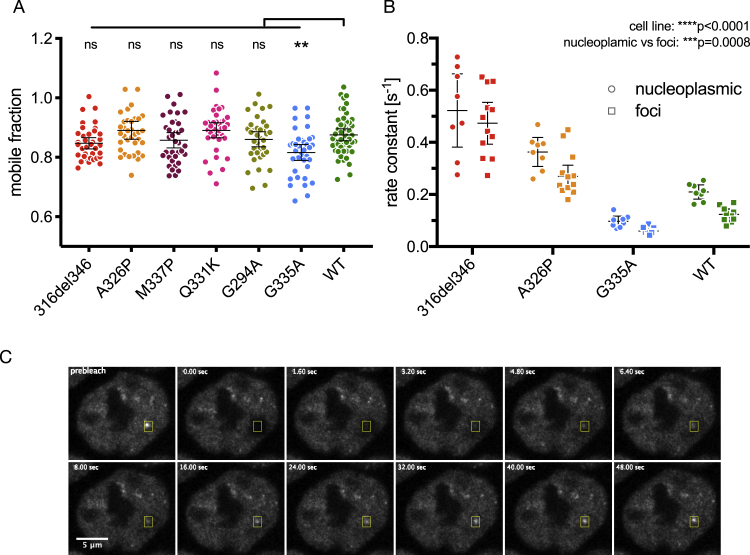
CR point mutants of TDP-43 have a gradient of *in vivo* condensation properties, related to Figure 3 A. Mobile fraction obtained from the plateau of the fitted exponential curve from FRAP experiments shown in Figure 3E. Mean ± 95%CI are shown for n (cells): 316del346 = 37, A326P = 36, M337P = 36, Q331K = 36, G294A = 36, G335A = 36, WT = 48. Significance was tested with Kruskal-Wallis test followed by Dunn’s Multiple Comparison Test (^∗∗^p adj. < 0.01). B. Rate constant of fluorescence recovery from each of the 316del346, A326P, G335A and WT GFP-TDP-43 cell lines, in foci-centered regions or the surrounding nucleoplasm obtained from FRAP experiments shown in Figure 3G. Mean ± 95%CI are shown for n (cells): 316del346 nucleoplasmic = 8, 316del346 foci = 12, A326P nucleoplasmic = 8, A326P foci = 12, G335A nucleoplasmic = 8, G335A foci = 12, WT nucleoplasmic = 8, WT foci = 10, 1 focus per independent cell. Significance was tested with Two-way ANOVA (cell line: ^∗∗∗∗^p < 0.0001, nucleoplasm versus foci: ^∗∗∗^p = 0.0008, interaction: not significant p = 0.6656). C. As in Figure S2D, representative image series and ROI from focus-centered FRAP experiments (Figure 3G) of GFP-TDP-43 WT cells.

Studies of purified TDP-43 CTD identified specific missense mutations that have variable effects on the helix-forming propensity of the CR, which are reflected by effects on LLPS (Conicella et al., 2016, 2020; Li et al., 2018). Therefore, we generated five TDP-43 HEK293 Flp-In cell lines with point mutations within or near the CR; we focus on these lines for the remaining experiments in this study (Figures 3A and 3B). The most perturbing mutation is A326P, which replaces the helix-promoting alanine with proline, disrupting the secondary structure (Conicella et al., 2016; Li et al., 2018). A slightly less disruptive mutation is M337P, which mimics the effects of an ALS-causing mutation (M337V); both mutations disrupt the CR helix-helix interactions and helical region extension to perturb LLPS (Conicella et al., 2016). The ALS-causing mutation Q331K is positioned within the CR helix and perturbs LLPS by disrupting helix-helix interactions without breaking the helical region (Conicella et al., 2016; McGurk et al., 2018). We also included the ALS-causing mutation G294A, located in IDR1 just upstream of the CR, which has been shown to only slightly decrease the tendency for higher-order interactions *in vitro* (Johnson et al., 2009). Finally, G335A has been shown to extend the CR helix and enhance LLPS (Conicella et al., 2020). All missense mutant constructs displayed nuclear localization, and their propensity for condensation, measured by foci count and FRAP mobility (see above), ranked between the WT and 316del346 (Figures 3C–3F and S3A). These experiments resulted in the expected trend of the strongest disruption by A326P, followed by M337P and then Q331K. In contrast, G294A weakly and G335A strongly promoted condensation.

The mutants with slower mobility displayed a larger number of nuclear foci, and so we assessed whether mobility was affected within the foci as well as in the nucleoplasm. We photobleached nuclear regions containing individual foci in the 316del346, A326P, G335A, and WT cell lines (Figure S3C). The three mutant constructs were chosen because they displayed faster or slower mobility compared with the WT. In general, the fluorescence recovery rate of bleached areas is slower for regions containing foci than in the surrounding nucleoplasm (Figures 3G and S3B; two-way ANOVA, p = 0.0008). The differences in mobility of the four constructs were recapitulated in foci-centered regions, demonstrating that mutations affect the mobility of TDP-43 in nuclear foci and the nucleoplasm. Thus, the mutants affect nuclear foci formation and mobility of TDP-43 in a graded fashion, ranging from disruptive (e.g., 316del346) to enhancing (e.g., G335A) effects, in line with observations from *in vitro* LLPS studies.

### CR-mediated condensation fine-tunes the RNA sequence preferences of TDP-43

**Figure 4:**
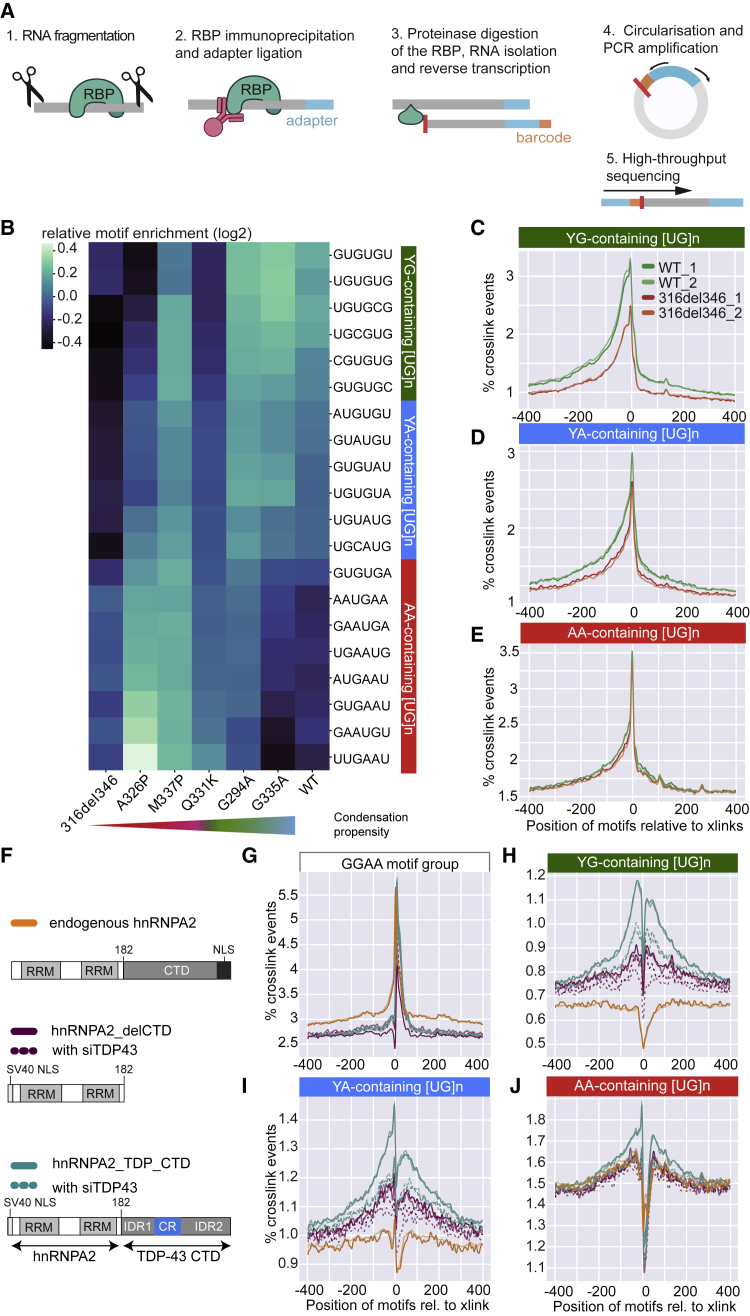
iCLIP reveals that condensation properties affect TDP-43 binding to specific RNA motifs (A) iCLIP involves UV-C crosslinking, cell lysis, RNA fragmentation by RNaseI, immunoprecipitation (IP) of crosslinked RBP-RNA complexes, and ligation to an infrared dye-labeled linker. After SDS-PAGE and transfer to a nitrocellulose membrane, RNA is released by proteinase K digestion, reverse transcribed, circularized, and PCR amplified. (B) The relative enrichment of the 20 6-mers that are most enriched across the “Mutants, low RNase experiment” iCLIP experiment (Table S1). The mean intronic motif enrichment of two replicates of each TDP-43 variant was normalized by the mean enrichment across all variants to define relative enrichment and plotted on the heatmap (log_2_ scale). The motifs were sorted based on the gradient of enrichment across TDP-43 variants and combined into three groups that are named according to the dominant sequence consensus: YG-containing [UG]n (green), YA-containing [UG]n (blue) or AA-containing [UG]n (red) (where Y indicates C or U). (C–E) Metaprofiles of YG-, YA-, or AA-containing [UG]n coverage around crosslink events in introns of replicates from the “RNase experiment" (Table S1). (F) hnRNPA2 domain map and design of its CTD deletion and TDP-43 fusion variants that were used for the “chimeraRBP-CLIP” experiment (Table S1). (G–J) Metaprofile of GGAA-type, YG-, YA-, or AA-containing [UG]n coverage around crosslink events in introns of replicates from the “chimeraRBP-CLIP” experiment, including samples with endogenous TDP-43 depletion (siTDP43).

**Figure S4:**
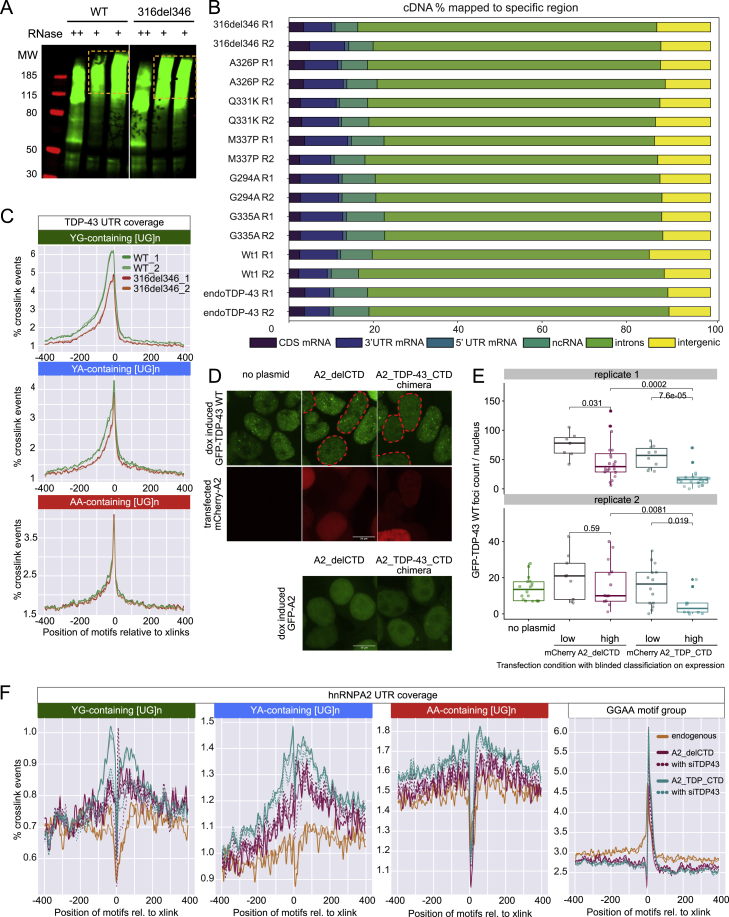
iCLIP reveals that condensation properties affect TDP-43 binding to specific RNA motifs, related to Figure 4 A. In this representative iCLIP experiment, the RNA/TDP-43 complex was visualized by Li-Cor scanning of nitrocellulose membrane, which detects the infrared adaptor that is ligated to the protein-RNA complexes. Shown here are WT (MW 85kDa) and 316del346 construct (MW 81kDa). The shift of the RNA-TDP-43 complex to higher molecular weight - highlighted by the orange box - is caused by the cross-linked RNA and ligated adaptor. RNase concentration was at 0.4 (+, low), 2 units (++, high) per 1 mL of lysate at 1mg/ml protein concentration and libraries were produced from the two low-concentration replicates. B. The number of unique cDNAs mapping to each region of transcriptome is shown for each replicate for the experiment shown in Figure 4B, and compared to the iCLIP with the endogenous TDP-43. C. Metaprofile of YG-, YA- or AA-containing [UG]n coverage around crosslink events in 3′UTRs of replicates from the ‘RNase’ iCLIP experiment. D. *Top*: Maximum z-projection images of HEK293 Flp-In cell lines expressing the indicated dox-inducible GFP-TDP-43 WT, transfected with mCherry-hnRNPA2-delCTD or mCherry-hnRNPA2-TDP-CTD. *Bottom*: HEK293 Flp-In cell lines expressing dox inducible GFP-hnRNPA2-delCTD or GFP-hnRNPA2-TDP-CTD. E. As in (D) Boxplot showing quantification of GFP-TDP-43 WT foci count per nucleus upon transfection with the indicated mCherry-hnRNPA2 construct. Nuclear segmentation of images from coverslips transfected with mCherry-hnRNPA2 were blinded, then the mCherry channel was classified as low or high expression manually, two replicate experiments. n (cells): replicate 1: A2-delCTD low = 8, A2-delCTD high = 19, A2-TDP-CTD low = 10, A2-TDP-CTD high = 21; replicate 2: no plasmid control transfection = 18, A2-delCTD low = 9, A2-delCTD high = 13, A2-TDP-CTD low = 14, A2-TDP-CTD high = 10. Significance was tested with t test. F. Metaprofile of GGAA-type motif, and YG-, YA-, AA-containing [UG]n around crosslink events in 3′UTRs of replicates from the ‘chimeraRBP-CLIP′ iCLIP experiment, including samples with endogenous TDP-43 depleted (siTDP43).

It remains unclear whether mutations that alter the CR-mediated condensation of TDP-43 also affect its RNA-binding properties. To tackle this, we performed crosslinking and immunoprecipitation (iCLIP) to obtain transcriptome-wide RNA binding profiles for WT TDP-43, the CR deletion (316del346), and each of the missense mutants (mutants experiment, Table S1) after 24 h of doxycycline induction (Figure 4A). The WT and 316del346 proteins gave similar signal intensities, indicating that loss of CR-mediated condensation does not affect the absolute amount of RNA binding (Figure S4A). We performed duplicate iCLIP experiments using each cell line after induction of the GFP-TDP-43 variants (mutants experiment, Table S1) and endogenous TDP-43 from uninduced cells. Computational analysis of crosslink sites in the iCLIP data revealed similar binding profiles for all GFP-TDP-43 variants and endogenous proteins, with most binding occurring at introns and 3′ UTRs (Figure S4B). We conclude that CR-mediated condensation of TDP-43 does not affect the general capacity of TDP-43 to bind RNA nor its ability to bind different types of RNAs.

To assess whether the point mutations affect TDP-43 binding at a more detailed, sequence-specific level, we quantified the prevalence of hexanucleotide (6-mer) motifs around intronic crosslink sites. We visualized the 20 most enriched 6-mers in a heatmap, comparing their relative enrichments for the WT and condensation-promoting G335A variant and the condensation-deficient A326P and 316del346 variants (Figure 4B). These top 20 6-mers contained one or more UG dinucleotides, indicating that many crosslinks occur around high-affinity UG repeats that are known to be bound by TDP-43 (Buratti et al., 2005; Tollervey et al., 2011). The WT and condensation-promoting G335A variant displayed binding preferences similar to all 20 6-mers. However, most condensation-deficient variants displayed decreased binding to a subset of 6-mers compared with the WT. The first group of 6-mers showed the most dramatic decrease among the condensation-deficient variants; they have the least divergence from the UG repeat ([UG]n), with a possible C-to-U replacement. We therefore refer to this motif group as YG-containing [UG]n (Y indicating pyrimidine). The second group had modestly decreased binding; these 6-mers diverge more from the UG repeat by replacing G with an A (such as UGUAUG), and we therefore refer to this motif group as YA-containing [UG]n. The third group did not display decreased binding; these 6-mers diverge most by interrupting the repetitive YRYRYR (pyrimidine/purine) pattern, most often with an AA dinucleotide (such as UGAAUG), and we therefore refer to this motif group as AA-containing [UG]n. It is striking that, even though the 6-mers were ranked by their relative enrichment across TDP-43 variants and not sequence content, they segregated into these three groups of motifs distinguished by the degree and type of divergence from the canonical UG repeat.

These observations were confirmed in additional iCLIP experiments comparing the WT and 316del346 variants at two RNase concentrations (Table S1). The distributions of the three motif types around intronic and 3′ UTR crosslink events showed that 316del346-binding was decreased at YG-containing [UG]n (Figures 4C and S4C) and to a lesser extent at YA-containing [UG]n (Figures 4D and S4C) but not at AA-containing [UG]n motifs (Figures 4E and S4C). Strikingly, the distribution profiles of YG- and YA-containing [UG]n motifs around crosslink events were very broad, whereas AA-containing [UG]n motifs were much more narrowly enriched. These findings demonstrate that the capacity of TDP-43 for CR-mediated condensation is required for its optimal binding to the broadly distributed YG- and YA-containing [UG]n but less important for binding to AA-containing [UG]n motifs.

### Homomultimeric interactions drive the CR-dependent RNA assembly

The *in vitro* LLPS effects indicate that the CR mutations change the capacity of TDP-43 for homomeric assembly. Additionally the CTD of TDP-43 is known to form heteromeric contacts with other proteins (Budini et al., 2014), but how mutations affect RNA binding *in vivo* remains unresolved. To probe the mechanism of CTD activity, we generated two variants of hnRNPA2, a protein with a domain structure similar to TDP-43, with which it has been reported to interact, although the direct protein-protein interaction appears to be weak (Buratti et al., 2005; Ryan et al., 2018). We generated an hnRNPA2 variant that lacked the CTD (hnRNPA2_delCTD) and a chimeric variant in which hnRNPA2′s CTD was swapped for that of TDP-43 (hnRNPA2_TDP_CTD; Figure 4F). The hnRNPA2 CTD has a C-terminal nuclear localization sequence (NLS) (Siomi and Dreyfuss, 1995), and therefore we added an N-terminal NLS to both variants to warrant nuclear localization. Surprisingly, neither variant localized to the nuclear TDP-43 foci, and coexpression of mCherry-hnRNPA2_TDP_CTD, but not mCherry-hnRNPA2_delCTD, actually led to a decreased number of TDP-43 foci when co-expressed with WT GFP-TDP-43 (Figures S4D and S4E). We then performed iCLIP experiments with both variants (chimeraRBP-CLIP, Table S1) as well as with endogenous hnRNPA2 with and without simultaneous siRNA depletion of the endogenous TDP-43. The metaprofiles showed that endogenous hnRNPA2 crosslinks strongly to its expected GGAA-type motifs at introns and 3′ UTRs (Huelga et al., 2012). This pattern was also seen for both variants (Figures 4G and S4F). Strikingly, we also found strong enrichment of the hnRNPA2_TDP_CTD variant on YG- and YA-containing [UG]n motifs that are bound by TDP-43 in a CR-dependent manner (Figures 4H, 4I, and S4F). Enrichment of the chimeric protein was seen up to 100 nt surrounding the TDP-43 binding motifs. Importantly, this enrichment was observed only when endogenous TDP-43 was present because its depletion decreased binding to the same level seen for the hnRNPA2_delCTD. In contrast, both hnRNPA2 variants showed less binding to the AA-containing [UG]n motif, which is bound by TDP-43 in a CR-independent manner, with only slight enrichment seen around this motif (Figures 4E, 4J, and S4F). ChimeraRBP-CLIP shows that the CTD-mediated homomultimeric contacts recruit the chimeric protein close to the RNA-binding regions of endogenous TDP-43.

### Three RNA features define the characteristics of binding-region condensates

**Figure S5:**
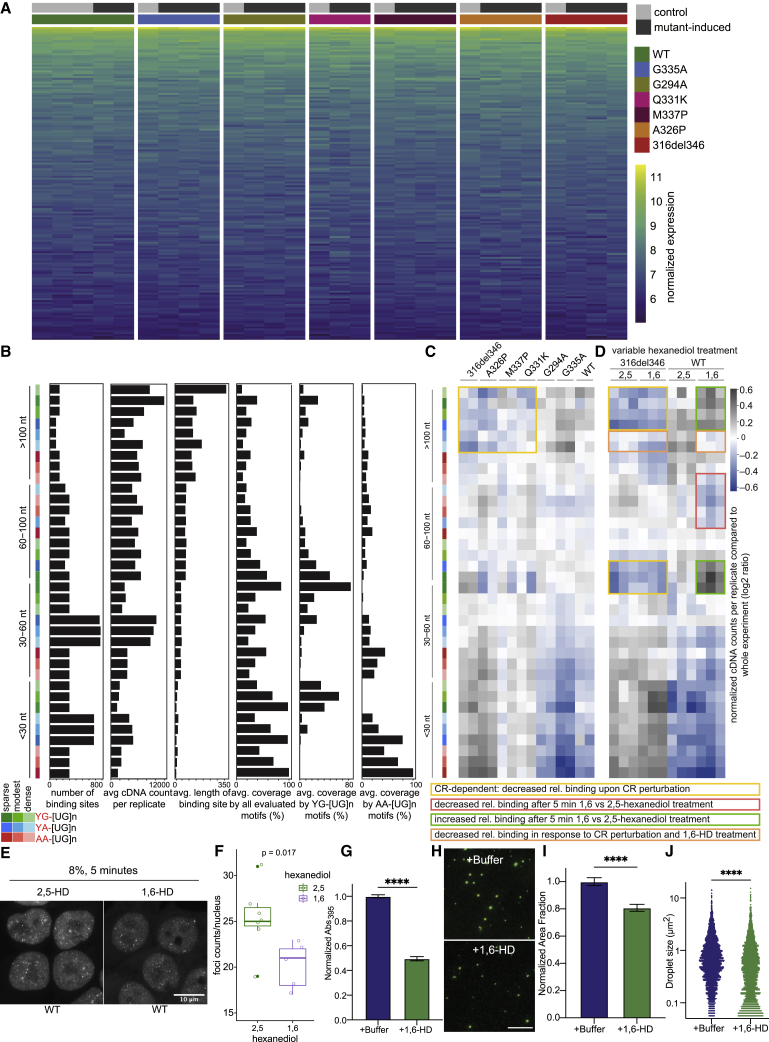
Three RNA features define the condensation-binding relationships, related to Figure 5 A. Normalized gene expression for the genes with regulated PAS (see Figure 6A) in control and after WT or TDP-43 variant induction. B. The features of each binding region class, which contribute to their classification. The 36 classes of binding regions are ordered as in Figure 5, and the following features are shown: the number of regions in each class, the average cDNA count per replicate in the ‘HD’ experiment (Table S1), average length of regions in each class, the average density of all evaluated motifs, and the average coverage of YG- or AA-containing [UG]n motifs. C. A separate experiment was analyzed as explained in Figure 5A of TDP-43 mutant lines at a lower RNaseI concentration (0.2 units per 1 mL of lysate) resulting in longer RNA fragments. D. A separate experiment was analyzed as explained in Figure 5A, containing WT TDP-43 and 316del346 triplicate samples pre-treated either with 1,6- or 2,5-HD. C and D are linked to Table S3 containing quantification of cDNA counts from CLIP samples overlapping with the binding regions, together with their genomic coordinates, region, gene id and gene names and derived classifications in groups by length, density and base content. E. Maximum z-projection of confocal z stacks of dox-induced GFP-TDP-43 WT in HEK Flp-In cells after incubation with 8% 2,5-HD or 8% 1,6-HD for 5 minutes. F. Quantification of foci counts in each segmented nucleus from confocal z stacks. n (counted cells): 2,5-HD = 7, 1,6-HD = 5; segmented from n (fields of view): 2,5-HD = 2, 1,6-HD = 2. Significance was tested with a Welch Two Sample t test (^∗^p = 0.017). G. Turbidity measurements on pre-formed TDP-43-MBP condensates show a reduction in turbidity after addition of 1,6-HD compared to addition of buffer (n = 3, two-tailed t test, p < 0.0001). H. Alexa488-labeled TDP-43-MBP (1:200 labeled:unlabeled) was used to image TDP-43-MBP condensates after addition of buffer or 1,6-HD. Scale bar, 10 μm. I. Decrease in area fraction of TDP-43-MBP condensates upon addition of 1,6-HD compared to addition of buffer (n= 15 images, two-tailed t test, p < 0.0001). J. Decrease in TDP-43-MBP condensate size upon addition of 1,6-HD compared to addition of buffer (n = 15 images, two-tailed t test, p < 0.0001).

**Figure 5:**
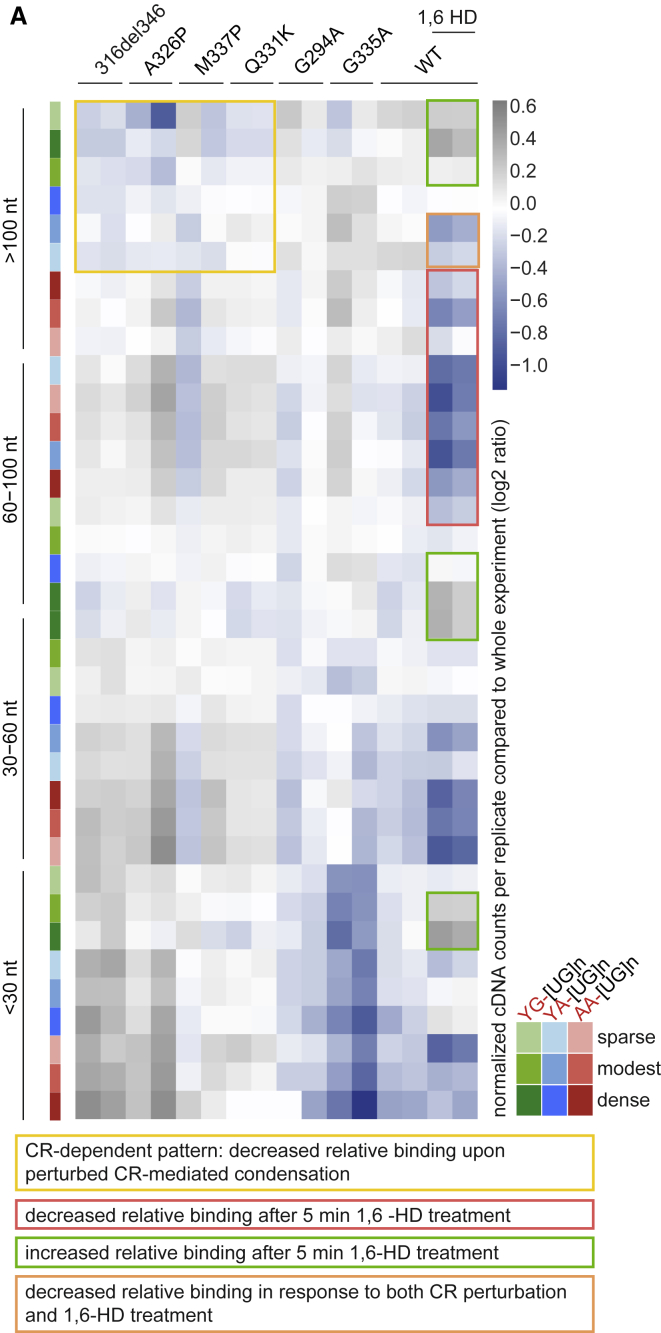
Three RNA features define the condensation-binding relationships (A) Binding regions were defined based on motifs proximal to crosslinks, and each region was allocated to one of 36 classes defined by the region length, motif density, and dominant motif type. The count of cDNAs falling into each class of binding regions from each iCLIP dataset was determined and normalized by the average cDNA count across all datasets within this experiment. Duplicate samples were obtained for each TDP-43 variant. Blue color represents depletion compared with the average, and gray represents enrichment. This is linked to Table S2 containing quantification of cDNA counts from CLIP samples overlapping with the binding regions together with their genomic coordinates, region, gene ID and gene names, and derived classifications in groups by length, density, and base content.

Because the CR mutations only disrupted binding to a subset of binding motifs, we reasoned that only a subset of the RNA-binding sites might require CR-mediated condensation for efficient binding to TDP-43. TDP-43 binds to clusters of motifs on endogenous RNAs, but the term “binding site” is sometimes used to refer to individual RNA motifs; therefore, we use the term “binding region” in the rest of this study, and we consider regions that are bound by TDP-43 in dependence of the CR helix-mediated condensation as types of “binding-region condensates.” To disentangle these features, we first defined the binding regions of TDP-43 by developing a computational approach (see Motif- and iCLIP-based binding region assignment in STAR Methods) to identify the top-ranking 6-mers that are located in close proximity to crosslinks and then grouped the motifs that are separated by up to 30 nt into 122,170 motif clusters. These regions were allocated into 36 classes by first placing them into 4 length classes, each of which was partitioned into three equally sized groups based on the density of motifs, and each of these was further divided into three equally sized groups based on the prevalence of YG-, YA- or AA-containing [UG]n motifs (Figure S5B). We found that induction of mutant variants of TDP-43 for the purpose of iCLIP did not detectably change gene expression of HEK cells, which simplified the analysis of binding trends at individual RNA sites (Figure S5A). To visualize the binding trends for each of the 36 classes of binding regions, we produced a heatmap showing the combined cDNA counts from iCLIP of WT, 316del346, and point mutants across all regions in each class. We highlighted the CR-dependent classes; i.e., those with relatively decreased cDNA counts in iCLIP of TDP-43 variants with perturbed CR-mediated condensation. These classes generally contain long binding sites (>100 nt; yellow outline in Figure 5A) with predominant YG- and YA-containing [UG]n motifs, suggesting that CR-dependent condensation contributes to efficient TDP-43 binding to regions containing extremely long clusters of specific motif types.

Next we analyzed the classes that do not show decreased binding among condensation-deficient CR-variants. Crosslinking of the CR-variants appears to increase at some of these classes, but this needs to be interpreted with caution because each class on the heatmap is internally normalized by the average binding across all variants for the sake of visualization; the apparent increase at CR-independent regions thus likely results from the decreased binding to CR-dependent regions. These CR-independent classes are shorter and more densely covered by motifs (Figure S5B, panel 4). The patterns of CR sensitivity were reproduced in three independent experiments (Figures 5A, S5C, and S5D) and were robust to variations in RNase activity, the aspect that is hardest to control in iCLIP experiments, as evident from results produced with medium (Figure 5A) or low-RNase conditions (Figure S5C) (mutants experiment, Mutants; low RNase experiment, Table S1). Among mutants, the relative changes in iCLIP binding correlated with the extent of perturbed condensation, as observed by imaging (Figure 3). The decrease in the relative binding to more than 100 nt versus less than 60 nt regions was strongest for 316del346 and A326P and milder with M337P and Q331K, and G294A had a similar iCLIP pattern as the WT protein. Conversely, G335A had increased binding to the more than 100 nt regions compared with the WT, which agrees with its increased condensation propensity. Importantly, multivalency was not a sufficient criterion explaining CR sensitivity because all classes of binding regions were highly multivalent, and the short regions were most densely covered by motifs (Figure S5B, panel 4). To highlight that multivalency alone is not the only RNA feature defining the binding-region condensates, we refer to the CR-dependent regions as “long-multivalent” for the sake of further discussion. A combination of length, motif type, and density characterizes the CR helix-mediated binding-region condensates, which assemble mainly on long multivalent regions with relatively low density of predominantly YG- and YA-containing [UG]n motif types.

Finally, we examined how 1,6-HD or 2,5-hexanediol (2,5-HD), which tends to be less disruptive to condensates (Alberti and Hyman, 2021), affect TDP-43 condensation in cells. 5-min treatment of cells with 8% 1,6-HD decreased the number of TDP-43 nuclear foci compared with 2,5-HD (Figures S5E and S5F). Likewise, at the pure protein level, 1,6-HD completely prevents LLPS of full-length TDP-43 (Mann et al., 2019) and dissolves preformed TDP-43 condensates (Figures S5G–S5J). We also produced iCLIP data from cells pre-incubated for 5 min with 8% 1,6-HD or 2,5-HD. We performed two iCLIP experiments where 1,6-HD treatment was compared with untreated cells (Figure 5A) or with 2,5-HD treatment (Figure S5D) (mutants experiment, HD experiment, Table S1); in both cases, 1,6-HD generally perturbed binding to long binding regions. The classes of binding regions affected by 1,6-HD were generally different from the CR-sensitive classes, which results mainly from the prevalence of different types and density of motifs in these classes (Figure 5A). 1,6-HD generally decreased binding to classes with more than 60 nt regions containing a relatively low density of predominantly AA- and/or YA-containing [UG]n motifs (red outline in Figure 5A) but increased binding to classes with predominantly YG-containing [UG]n motifs (green outline in Figure 5A). In fact, condensation-deficient CR variants and 1,6-HD treatment have an opposing effect at long regions with predominant YG- or dense YA-containing [UG]n motifs (yellow versus green outline in Figure 5A). Nonetheless, one class showed a similar sensitivity to CR variants and 1,6-HD treatment: more than 100 nt regions containing a relatively low density of predominantly YA-containing [UG]n motifs (orange outline in Figure 5A). 1,6-HD treatment generally perturbed TDP-43 binding to different classes of binding regions than CR mutations, depending on the length of the regions and the density and type of binding motifs they contain.

### TDP-43 condensates form on individual binding regions

**Figure 6:**
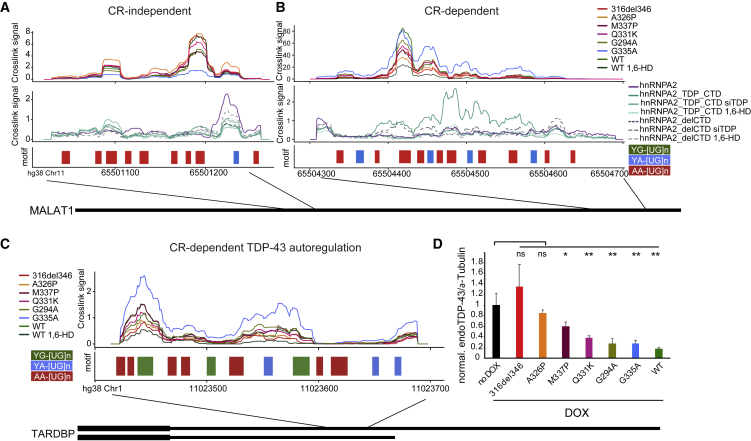
TDP-43 shows distinct condensation-dependent binding, and CR mutants have defects in autoregulation (A) Mapping of TDP-43 iCLIP data onto Malat1 non-coding RNA. Two replicates were summed, and iCLIP data were normalized and converted into smoothed lines using the rollmean function with a window size of 20 and mapped to a 250-nt-long regions on the ncRNA Malat1 (hg38 chr11:65501021-65501271:+) with CR-independent binding behavior. A crosslinking signal derived from the following two different iCLIP experiments is shown. Top panel: CR mutant TDP-43 variants. Center panel: hnRNPA2 constructs as described in Figure 4F. Bottom panel: the assigned binding regions colored according to their motif bias: YG-, YA-, and AA-containing [UG]n in green, blue, and red, respectively. (B) As in (A) for a CR-dependent and 1,6-HD-sensitive binding region on a 400-nt-long region of the ncRNA Malat1 (hg38 chr11:65504300-65504700:+). (C) As in (A) for a CR-dependent and 1,6-HD-sensitive region in the 3′ UTR of the endogenous TARDBP RNA (hg38 chr1:11023414-11023698:+). (D) Quantification of the western blot analysis of the endogenous TDP-43 levels after 2 days of induction of each of the GFP-TDP-43 variants; see the corresponding western blot in Figure S6B. Statistical significance of n = 3 was calculated using Student’s t test with ^∗^p < 0.05, ^∗∗^p < 0.01.

**Figure S6:**
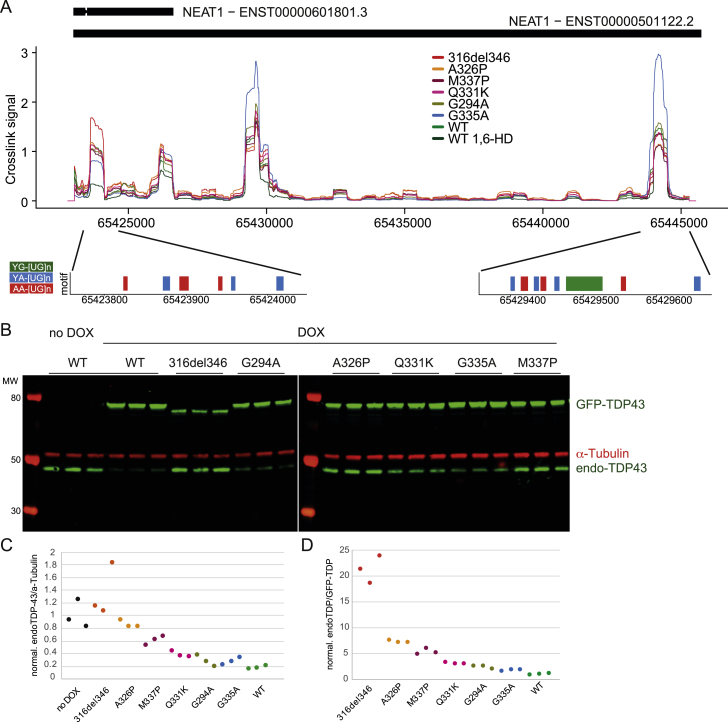
TDP-43 shows distinct condensation-dependent binding, and CR mutants have defects in autoregulation, related to Figure 6 A. Mapping of TDP-43 iCLIP data onto Neat1 ncRNA. Two replicates were summed and iCLIP data was normalized and converted into smoothed lines using rollmean with window size of 500 to two 22kB long regions on the ncRNA Neat1 with CR-dependent and -independent binding behavior. Crosslinking signal was derived from CR mutant TDP-43 variants. The bottom panel shows a motif-based binding site assignment where 300nt regions are colored according to their motif bias: YG-, YA-, AA-containing [UG]n in green and blue and red, respectively. B. Western blot analysis of expression level of the endogenous TDP-43 and GFP-TDP-43 variants after 2 days of induction with doxycycline (DOX), as determined by the anti-TDP-43 antibody. Data was compared to no DOX level of the endogenous TDP-43 protein and loading was normalized by alpha-tubulin as a loading control. C. Quantification of western blot analysis of the endogenous TDP-43 levels after two days of induction of each of the GFP-TDP-43 variants. Each sample replicate was normalized by alpha-tubulin as a loading control. D. Same as in (C) except, each sample replicate was normalized by GFP-TDP-43 variant expression.

To date, studies of RNA-condensation relationships have been done with full-length RNAs (Langdon et al., 2018; Lee et al., 2020; Maharana et al., 2018). Therefore, it remains unclear whether RBP condensation can involve individual binding regions. We addressed this question by analyzing two abundant long non-coding RNAs (lncRNAs) that contain multiple binding regions with large numbers of iCLIP cDNAs: *NEAT1* and *MALAT1*. These lncRNAs participate in cross-regulation with TDP-43 and are differentially bound in brain tissue from individuals with FTD (Modic et al., 2019; Nguyen et al., 2020; Tollervey et al., 2011), and NEAT1 has been found to influence the phase separation propensity of bound RBPs (Maharana et al., 2018). Both RNAs contain multiple TDP-43 binding regions, with some regions displaying CR-dependent binding patterns, whereas others were CR independent. In *MALAT1*, two primary binding regions were ∼9 kb apart on the RNA. The CR-dependent region showed greatly reduced binding of 316del346 compared with the WT, with the expected gradient of binding loss across the point mutants (Figure 6B). The CR helix-disrupting mutations and 1,6-HD led to dramatic binding decreases. Conversely, the CR-independent region did not show decreased binding when the CR helix was mutated or upon 1,6-HD treatment (Figure 6A). The CR-dependent region was more than 300 nt long and had broad binding consistently across the whole region, whereas the CR-independent region was nearly 150 nt long and had discrete, short binding peaks. Strikingly, the hnRNPA2_TDP_CTD chimeric protein strongly increased its binding to the CR-dependent region but not the CR-independent region, and this increased binding was lost upon 1,6-HD treatment or upon depletion of the endogenous TDP-43 (Figures 6A and 6B, bottom panel). In *NEAT1*, we identified four binding regions with distinct binding behaviors (Figure S6A). The first two binding regions at the 5′end of *NEAT1* showed sensitivity to 1,6-HD treatment but not to CR helix mutations (Figure S6A). The third and fourth binding regions, in contrast, showed moderate reliance on CR for binding, no sensitivity to 1,6-HD treatment, and enhanced binding of the hypercondensing variant G335A. The insensitivity to 1,6-HD is in agreement with an imaging study showing that paraspeckles, which are scaffolded by *NEAT1*, withstand 1,6-HD treatment (Yamazaki et al., 2018). We conclude that TDP-43 does not assemble via a uniform mechanism on RNAs with multiple binding regions but, rather, that condensation of TDP-43 takes place on individual RNA-binding regions.

### The TDP-43 3′ UTR binding region condensate mediates autoregulation

As described above, one of the regions in *MALAT1* was perturbed by CR mutations as well as by 1,6-HD (Figure 6B). Fascinatingly, another doubly sensitive binding region was found within the 3′ UTR of the endogenous *TDP-43* mRNA itself (Figure 6C). This binding region has been shown previously to mediate autoregulation so that binding of TDP-43 to its own mRNA changes the processing of the 3′ UTR and, thus, decreases mRNA abundance (Ayala et al., 2011; Polymenidou et al., 2011). Therefore, we monitored TDP-43 expression by western blotting after 2 days of doxycycline-induced expression of GFP-TDP-43 variants in the presence of endogenous TDP-43 (Figures 6D and S6B). Strikingly, induction of the 316del346 deletion abrogated the autoregulatory capacity, the helix-breaking mutations A326P and M337P almost completely lost the capacity, and Q331K had 50% lower capacity compared with the WT construct. The expression level of 316del346 is lower in comparison with all other constructs, which likely contributes to its reduced autoregulation capabilities, but even when normalizing endogenous TDP-43 by the transgene protein level, 316del346 still has the lowest autoregulatory capacity (Figure S6D). Further, helix-modifying mutations have similar expression levels as the WT, but the gradient of their loss in autoregulation capacity was correlated to the gradient of binding loss as defined by iCLIP. These findings show that CR helix-mediated condensation is essential for efficient assembly of TDP-43 on the 3′ UTR of its own mRNA and, therefore, its autoregulation.

### Condensation is required for TDP-43 function at a subset of its regulated mRNAs

The autoregulation effect revealed that CR-mediated condensation contributes to the regulatory function of TDP-43. Conversely, condensation-deficient TDP-43 variants in IDR1/IDR2 can retain their function, regulating splicing of a few RNAs (Schmidt et al., 2019). TDP-43 regulates a broad range of 3′ end processing events (Modic et al., 2019; Rot et al., 2017), and therefore we investigated whether condensation-deficient CR variants are deficient in regulating any of these events. 24 h after transfecting siRNAs to deplete the endogenous TDP-43, we induced the siRNA-resistant GFP-TDP-43 variants and collected cells 24 h afterward. Total RNA was isolated from cells and used for 3′ mRNA sequencing.

**Figure S7:**
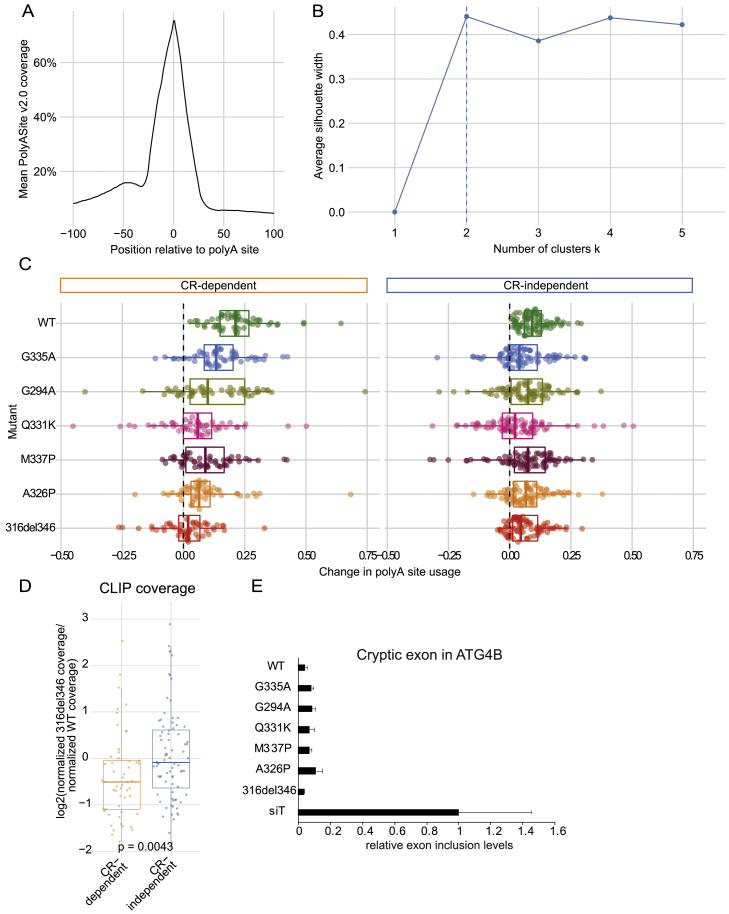
Regulation of a subset of PAS is sensitive to CR mutations, related to Figure 7 A. The distribution of PAS from PolyASite 2.0 (Herrmann et al., 2020) around PAS defined from the 3′ end sequencing data. B. The optimal number of k-medoid clusters assessed using the average silhouette method. C. Related to Figure 7A: distribution of the relative change in dPAU (normalized such that WT is always positive) for each gene upon rescue by WT TDP-43 or each variant. Genes have been clustered according to their CR-dependence. D. The ratio of total iCLIP cDNA counts for helix-disrupting (316del346 and A326) normalized against helix-preserving variants (G335A and WT TDP-43) in the region between the proximal and distal PAS for CR-dependent and CR-independent genes. Statistical difference within each group was assessed with a Mann-Whitney test. E. qPCR quantification of the change in cryptic exon usage in the ATG4B gene after expression of TDP-43 variants in combination with siRNA mediated depletion of the endogenous TDP-43 (siT) (n= 3). This cryptic exon has very low expression in Hek-293 cells and this results in qPCR Ct values of 29 versus 25 after TDP-43 depletion.

**Figure 7:**
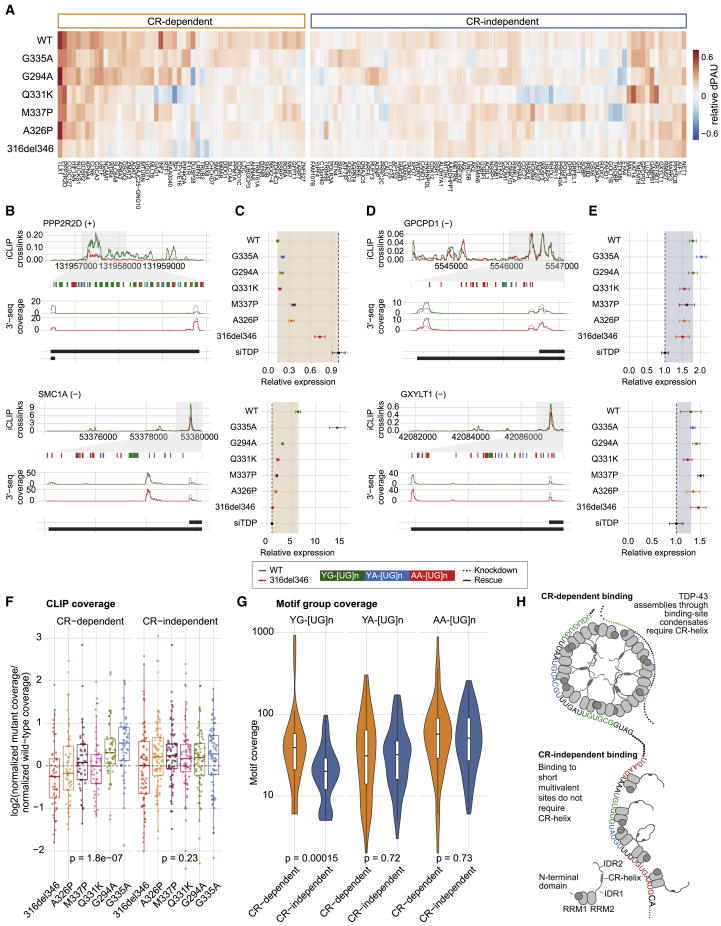
Regulation of a subset of poly(A) sites (PASs) is sensitive to CR mutations (A) The change in usage or delta polyA site usage (dPAU) for the representative PAS in each gene upon rescue by WT TDP-43 or each variant (Table S4). The dPAU for each PAS is normalized so that the WT change is always positive. Red shades indicate rescue in the same direction as the WT and blue shades in the opposite direction; white indicates no rescue. Genes are clustered according to their CR dependence. (B) Top panel: the normalized 316del346 and WT iCLIP coverage tracks (combining all 2,5-HD replicates for each condition) are shown between the proximal (left) and distal (right) PASs that show a regulation pattern that is sensitive to CR mutations (CR-dependent PAS). Center panel: binding motifs in the key binding region (shaded in gray) are magnified and colored by motif type. Bottom panel: the normalized 3′ seq coverage tracks (merging all replicates) for knockdown and rescue with the 316del346 variant and WT. (C) qPCR quantification of the change in PAS usage after expression of TDP-43 variants after knockdown of the endogenous TDP-43 (siT), showing the ratio of the use of distal versus proximal PASs. The shaded region highlights the magnitude of WT rescue over knockdown (n = 3, t test, p = 5.97 × 10^−10^ [PPP2R2D] and 1.68 × 10^−10^ [SMC1A]). (D) As in (B) for PASs that are regulated with similar efficiency by all TDP-43 variants regardless of CR mutations (CR-independent PASs). (E) As in (C) for CR-independent PASs. The shaded region highlights the magnitude of WT rescue over knockdown (n = 3, t test, p = 6.97 × 10^−8^ [GPCPD1] and 0.0158 [GXYLT1]). (F) The ratio of total iCLIP cDNA counts for each TDP-43 variant in the region between the proximal and distal PAS for CR-dependent and CR-independent genes and the ratio of total iCLIP cDNA counts for each TDP-43 variant normalized against WT TDP-43 (combining both biological replicates for each condition). Statistical difference within each group was assessed with an ANOVA. (G) The number of nucleotides covered by TDP-43 bound YG-, YA-, and AA-containing [UG]n motifs in the region between the proximal and distal PASs for CR-dependent and CR-independent genes. Statistical difference was assessed with a Mann-Whitney test. (H) Each RRM domain of TDP-43 recognizes only 4–6 nt in a sequence-specific manner, and TDP-43 binds highly multivalent RNA regions in cells. Condensation-deficient variants of TDP-43 have a decreased capacity to bind a subset of these regions, called “CR-dependent regions”; these tend to be more than 100 nt long and contain a medium density of predominantly YG- and YA-containing [UG]n motifs. The schematic highlights the likely role of CR helix-mediated homomeric interactions in enabling condensation of TDP-43 molecules at a relatively high density on the long CR-dependent regions. Such condensation is regional because long RNAs can contain CR-dependent and CR-independent regions. The conformation of contacts as shown here is purely schematic.

To analyze the capacity of CR variants to rescue appropriate 3′ end processing, we first derived a common atlas of poly(A) sites (PASs) using data from all experiments which enabled us to characterize unannotated sites. There was a good overlap with annotated sites in the PolyASite 2.0 atlas (Herrmann et al., 2020; Figure S7A). We then quantified the usage of each PAS with DRIMseq (Nowicka and Robinson, 2016) to assess the changes in ratios of PAS usage in each gene between siRNA knockdown treatment of each cell line and induction of the corresponding TDP-43 variant. This approach identified 200 genes with statistically significant changes (adjusted p < 0.05; likelihood ratio test with Benjamini-Hochberg correction) in PAS usage upon induction of at least one TDP-43 variant. Filtering those sites with a 10% or less change in PAS usage across variants left 166 genes (STAR Methods; Figure 7A).

Next we defined similarly regulated clusters of 136 of the 166 significant genes for which PAS usage could be determined for every rescue condition using partitioning around medoids. The silhouette method showed that the data optimally clustered into two groups (Figure S7B). The patterns of change in PAS usage for each distinct group reflected the capacity of each TDP-43 variant to regulate PAS usage relative to WT TDP-43. For the 82 CR-independent mRNAs, condensation-deficient TDP-43 variants (316del346, A326P, and M337P) were able to regulate PAS usage in the same way as the WT, but for the 54 CR-dependent mRNAs, this capacity was significantly impaired (Figures 7A and S7C). As supported by qPCR analysis, CR-dependent mRNAs displayed a gradual decrease in functionality from WT and G335A to the condensation-deficient variants, especially the A326P and the CR deletion (Figures 7B, 7C, and S7C). In contrast, CR-independent mRNAs displayed a functionality for all or most condensation-deficient variants to a similar level as seen for the WT protein (Figures 7D, 7E, and S7C).

To assess whether changes in the functionality of TDP-43 variants could be ascribed to changes in their RNA binding, we examined the iCLIP data on the 3′ UTRs of both mRNA classes. Binding of the condensation-deficient variants (316del346 and A326P) relative to condensation-preserving variants (WT and G335A) was significantly decreased in CR-dependent mRNAs (p = 0.0043) but not CR-independent mRNAs (Figure S7D). Moreover, the mutant variants of TDP-43 exhibited graded changes in binding to CR-dependent mRNAs that correlated with their condensation capacity (p = 1.8 × 10^−7^), with the strongest loss of binding seen for the A326P mutant, similar to 316del346 (Figure 7F). In contrast, TDP-43 variants did not significantly differ in their binding to CR-independent mRNAs (p = 0.23; Figure 7F). 3′ UTRs of CR-dependent mRNAs had a significantly increased number of YG-containing [UG]n (p = 0.00015), but not YA-[UG]n or AA-[UG]n, compared with CR-independent mRNAs (Figure 7G). These findings indicate that TDP-43 condensation is required for regulation of a subset of its mRNA partners. We observed a clear relationship between the condensation capacity of TDP-43 variants *in vitro* and *in vivo* (Figures 1, 2, and 3) and the capacity for iCLIP binding (Figures 4 and 5) and regulation (Figures 6 and 7) of a subset of RNA partners.

## Discussion

Our study introduces an integrative approach across a broad range of methods to study RNP condensation, including the RBPchimera-CLIP approach and bioinformatics tools to assign and classify bound RNA regions, to find that, by promoting homomeric assembly of TDP-43 on specific RNA regions, the CR helix selectively modulates the RNA binding specificity of TDP-43. Notably, CR helix mutants perturb TDP-43 condensation at multiple scales to a similar extent, including LLPS, nuclear mobility and foci formation, formation of binding-region condensates, and regulation of RNA processing, indicating that they are driven by the same homomultimerization forces. The binding-region condensates are promoted by a relatively low density of specific motif types that are dispersed over long RNA regions (generally more than 100 nt). Such condensation-dependent assembly on long multivalent regions selectively contributes to the regulatory capacity of TDP-43. TDP-43 contains two tandem RNA recognition motifs (RRMs) that have been shown to recognize only 4- to 6-nt-long motifs (Lukavsky et al., 2013); thus, it is plausible that CR-mediated condensation brings together a large number of RRMs to increase their combined binding avidity to the long-multivalent regions (Figure 7H). We have resolved the salient RNA features of binding-region condensates that help explain their role in selective RNP assembly and regulation of RNA networks.

Transcriptome-wide changes in RNA processing have been observed in post-mortem human tissue and in animal models of mutant TDP-43, and a decreased capacity of mutant TDP-43 to autoregulate its own expression has been observed in a mouse model of the Q331K ALS mutation, which increases the levels of TDP-43 protein (White et al., 2018), but the mechanism underpinning the impaired autoregulation remained unclear. Existing models of changes in the regulatory capacity of TDP-43 propose generic cytoplasmic gain and nuclear loss of function as candidates for ALS-causing mechanisms (Tziortzouda et al., 2021). Instead, we now find that the changes in function can be selective because various CR mutations lower the ability of TDP-43 to bind subclasses of binding regions while retaining binding and regulation of other RNAs. Thus, when perturbed condensation of TDP-43 decreases its capacity for autoregulation, gradually increasing its abundance, it could affect different RNAs in variable ways depending on whether the mutant TDP-43 can efficiently bind and regulate the RNA. At a later stage of ALS progression, the general activity of TDP-43 drops because of its mislocalization or aggregation, affecting all of its RNA partners. For example, qPCR analysis of the cryptic exons in ATG4B, which is derepressed upon TDP-43 depletion, showed that CR mutants and CR deletion were just as efficient in repressing this exon as the WT protein (Ling et al., 2015; Figure S7E). The 5′ splice site of the ATG4B cryptic exon is flanked by a 70-nt-long UG microsatellite repeat characteristic of CR-independent binding because it is very dense and less than 100 nt. Thus, the characteristics of the TDP-43-binding regions and the results of our qPCR analyses indicate that cryptic exons such as in ATG4B may not be directly affected by CR mutations but could become deregulated at the stage when nuclear clearance of TDP-43 starts to contribute to the disease process.

As further insight into the regulated RNA network, we identified 136 mRNAs with PAS switches, four of which are associated with ALS or TDP-43 regulation, including the core stress granule protein G3BP1, which is essential for granule formation in response to specific stressors (Guillén-Boixet et al., 2020; Yang et al., 2020). Depletion of TDP-43 promotes expression of the longer G3BP1 3′ UTR isoform, which is believed to be translated less efficiently and, thus, could decrease G3BP1 abundance (Sidibé et al., 2021). TDP-43 also regulates processing of Gemin6, a component of the Gem protein complex dysregulated in spinal muscular atrophy (Cauchi, 2014), and of CSNK1D, encoding one of the Ser/Thr Kinase CK-1 isoforms, which can phosphorylate up to 29 sites in TDP-43, some of these in response to stress (Kametani et al., 2009). Finally, TDP-43 regulates processing of KPNB1, encoding karyopherin β1, a nuclear-import receptor that counteracts the cytoplasmic accumulation of TDP-43 and antagonizes TDP-43 fibrillization (Guo et al., 2018; Nishimura et al., 2010). Our study provides insights into unprecedented feedback loops that could drive TDP-43 pathogenesis through aberrant phosphorylation, cytoplasmic mislocalization, and aggregation of TDP-43, the hallmarks of ALS (Tziortzouda et al., 2021).

It has been shown that RNAs can have specific effects on condensation properties of RBPs (Loughlin et al., 2021; Maharana et al., 2018; Mann et al., 2019; Sanders et al., 2020), but whether these effects are mediated by full-length RNA molecules or by specific binding regions has remained unclear. Several studies of splicing regulation provide evidence that variable condensation of RNPs might be able to selectively modulate RBP activities at subsets of RNA-binding sites (Ule and Blencowe, 2019). We identified long RNAs that contain CR-dependent and independent TDP-43 binding regions. The CR-independent regions are generally shorter and contain a more dense arrangement of motifs with enrichment of the UGAAUG-type motifs (Figure 7H). This motif is precisely aligned to the TDP-43 crosslink sites (Figure 4E), which agrees with structural studies that have shown that the two adenosines of the UGAAUG motif can anchor TDP-43 on the RNA by acting as a bridge between the β2-β3 loops of the two RRM domains (Lukavsky et al., 2013). Conversely, more than 100 nt long regions with a more dispersed arrangement of predominantly YG- and YA-containing [UG]n motifs tend to form CR-dependent binding-region condensates (Figure 7H). The more than 100 nt long regions with a sparser arrangement of predominantly AA-containing [UG]n are generally perturbed by 1,6-HD treatment rather than CR mutations. Interestingly, a small subset of binding regions (especially those enriched in YA-containing [UG]n) are sensitive to both types of condensation perturbations, as we show in the case of the MALAT1 and the 3′ UTR of *TARDBP* mRNA.

By showing that the effects of TDP-43 condensation are specific to subsets of binding regions, our model helps explain the findings of past studies that examined a few RNA partners of TDP-43 to conclude that condensation mediated by various CTD regions is or is not required for the function of TDP-43 in RNA processing (Budini et al., 2014; Conicella et al., 2020; Roczniak-Ferguson and Ferguson, 2019; Schmidt et al., 2019). Given that distinct RNA features were enriched at regions affected by CR mutation versus 1,6-HD treatment, one could speculate that distinct types of condensation-promoting interactions might be required at distinct classes of long-multivalent RNA regions. Many types of homomeric contacts have been reported to contribute to TDP-43 condensation. For instance, the 1,6-HD effect has been linked to the role of π-π interactions of aromatic residues in IDR1 and IDR2 that promote the LLPS of TDP-43 (Schmidt et al., 2019). In addition to the CR helix, various portions of the CTD can form highly polymorphic steric zippers, dagger-shaped folds, and R-shaped fibrils that could also mediate reversible or irreversible aggregation (Tziortzouda et al., 2021). The importance of homomeric CTD-mediated interactions in RNA recruitment is directly demonstrated by our iCLIP study of a chimeric hnRNPA2-TDP-CTD protein, which requires the endogenous TDP-43 to be present to bind to UG-rich RNA regions. Interestingly, this recruitment does not appear to exclusively occur within the foci because we do not observe any enrichment of the chimeric protein in the foci. Moreover, it has been shown that RNA binding makes TDP-43 less prone to macroscopic phase separation (Mann et al., 2019; Yu et al., 2021), and we find that, in addition to its effects on the foci, CR-mediated condensation also slows the nucleoplasmic mobility of TDP-43. These findings imply that region-specific RNA recruitment might be mediated by molecular-scale condensates of TDP-43 into nanometer-sized assemblies.

Multivalency is known to be an essential feature of RNA molecules that support condensation of bound RBPs, especially those containing IDRs (Gueroussov et al., 2017; Loughlin et al., 2021; Lyon et al., 2021; Ule and Blencowe, 2019), and several DNA-binding proteins have also been shown to require IDRs for binding to broad DNA regions (Brodsky et al., 2020). We now find that condensation selectively modulates TDP-43 assembly on long multivalent regions; thus, it will be of interest to also investigate such selective condensation for other proteins. The condensation properties of RBPs can be modified by mutations in disease and evolution (Lyon et al., 2021), including in the coacervate model of early RNP evolution (Astoricchio et al., 2020; Drobot et al., 2018; Poudyal et al., 2018), and by post-translational modifications in response to signaling pathways, cellular stress, or aging-related changes (Alberti and Hyman, 2021; Chong and Forman-Kay, 2016). Moreover, ongoing efforts are identifying therapeutic approaches that modulate condensates in neurodegeneration (Alberti and Hyman, 2021; Wheeler, 2020), and it will be important to assess whether such approaches might also selectively affect the functionality of affected complexes at the molecular scale. Our findings thus open the possibility that changes in RNP condensation could remodel RNA networks with high selectivity in development, disease, and evolution.

### Limitations of study

We find that CR-mediated condensation of TDP-43 is not needed for binding to all multivalent RNA regions, and its role additionally depends on the length of multivalent regions and the type and density of motifs in these regions. Although these features represent general trends of the whole class of CR-sensitive binding regions, there are variations between individual CR-sensitive RNA regions, and additional features may play a role. For example, RNP condensates can also be modulated by RNA structure, RNA-RNA contacts (Tauber et al., 2020), and proximal binding sites of multiple different RBPs (Attig et al., 2018). The CR-dependent and -independent RNA regions might differ in the structural arrangement of the RNA regions in complex with TDP-43; for example, so that the CR helix forms only in the first case. It will be particularly interesting to study with purified components how the presence of various RNAs affects the size, shape, and organization of TDP-43 condensates. Moreover, CR-dependent regions are long and contain highly dispersed TDP-43 binding motifs; thus, much of their sequence is available for binding to other RBPs. As a result, TDP-43 might face increased competition with other RBPs at the CR-dependent regions and require its CR-mediated condensation to outcompete (or coassemble with) these RBPs. Further *in vitro*, bioinformatics, and functional experiments will be needed to resolve these hypotheses.

## STAR★Methods

### Key resources table

**Table:** 

REAGENT or RESOURCE	SOURCE	IDENTIFIER
**Antibodies**		

Rabbit anti-TDP-43 Antibody	Proteintech	Cat#10782-2-AP, RRID:AB_615042
Mouse anti-α-Tubulin Antibody	Sigma	Cat# T5168, RRID:AB_477579
Mouse anti-FLAG Antibody	Sigma	Cat#F1804, RRID:AB_262044
Rabbit anti-GFP Antibody	Abcam	Cat# ab290, RRID:AB_303395
IRDye 800CW Donkey anti-Rabbit IgG (H + L)	Li-Cor	Cat#926-32213, RRID:AB_621848
IRDye 680RD Goat anti-Mouse IgG (H+L)	Li-Cor	Cat#926-68070, RRID:AB_10956588

**Bacterial and virus strains**		

NEB 5-alpha Competent *E. coli* (High Efficiency)	New England Biolabs	Cat#C2987I

**Chemicals, peptides, and recombinant proteins**		

GFP-TRAP_MA beads	Chromotek	Cat#gtma-10
Protein G Dynabeads	Dynal	Cat#10004D
Protein A Dynabeads	Dynal	Cat#10002D
BSA	NEB	Cat#B9000S
DC Protein Assay Kit	Biorad	Cat#5000111
Phusion High-Fidelity DNA Polymerase (2 U/μL)	Thermo Fisher Scientific	Cat#F530L
10 mM dNTP Mix	Thermo Fisher Scientific	Cat#18427088
UltraPure Agarose	Thermo Fisher Scientific	Cat#16500500
Kanamycin Sulfate	Thermo Fisher Scientific	Cat#BP906-5
Ampicillin Sodium Salt	Sigma-Aldrich	Cat#A0166-5G
2,5-HEXANEDIOL, 99%,	Sigma-Aldrich	Cat#H11904-50G
1,6-Hexanediol	Sigma-Aldrich	Cat#240117-50G
Fast SYBR Green Master Mix	Thermo Fisher Scientific	Cat#4385612
TRIzol reagent	Thermo Fisher Scientific	Cat#15596206
VECTASHIELD	Vector labs	Cat#H-1000
Lipofectamine 2000 Transfection Reagent	Thermo Fisher Scientific	Cat#11668019
Lipofectamine RNAiMAX Transfection Reagent-1.5 mL	Thermo Fisher Scientific	Cat#13778150
AMPure XP, 5 mL	Agencourt	Cat#A63880
RIPA Buffer	Sigma-Aldrich	Cat#R0278-50ML
cOmplete(TM) Protease Inhibitor Cocktail	Sigma-Aldrich	Cat#11697498001
NuPAGE Novex 4-12% Bis-Tris Protein Gels, 1.0 mm, 10 well	Thermo Fisher Scientific	Cat#NP0321BOX
Puromycin	Takara Clontech	Cat#631305
Blasticidin S HCl	ThermoFisher Scientific	Cat#R21001
Hygromycin B (50mg/ml)	ThermoFisher Scientific	Cat#10687010
Doxycycline hydrate	Sigma-Aldrich	Cat#D9891
Zeocin Selection Reagent	Thermo Fisher Scientific	Cat#R25001
Novex TBE Gels, 6%, 10 well-1 box	Thermo Fisher Scientific	Cat#EC6265BOX

**Critical commercial assays**		

SuperScript II Reverse Transcriptase	Thermo Fisher Scientific	Cat#18064014
Zero Blunt TOPO PCR Cloning Kit for Sequencing, with One Shot TOP10 Chemically competent E.coli	Thermo Fisher Scientific	Cat#K2875J10
QuantSeq 3′ mRNA-Seq Library Prep Kit FWD for Illumina	Lexogen	Cat#015.2x96
QuantSeq-Flex First Strand Synthesis Module	Lexogen	Cat#SKU: 026.96
Fast SYBR Green Master Mix	Thermo Fisher Scientific	Cat#4385612
Maxwell RSC simplyRNA Cells Kit	Promega	Cat#AS1390

**Deposited data**		

TDP-43 variant iCLIP in Hek-293	This study	Sequencing data deposited at EBI ArrayExpress under accession number "ArrayExpress: E-MTAB-9436"; Raw data processed on the iMaps webserver: https://imaps.goodwright.org/collections/868/
pAseq data	This study	Sequencing data deposited at EBI ArrayExpress under accession number "ArrayExpress: E-MTAB-9410"

**Experimental models: Cell lines**		

Human: HEK293T cells	European Collection of Authenticated Cell Cultures (ECACC)	12022001

**Oligonucleotides**		

Stealth RNAi siRNA Negative Control, Med GC	Thermo Fisher Scientific	Cat#12935300
TARDBP Stealth siRNA	Thermo Fisher Scientific	Cat#HSS177422
TDP43delCRrev	This study	CGCACCAAAGTTCATCCCACCACCCATA
TDP43delCRfw	This study	TCAGGCCCATCGGGTAATAACCAAAACCAAG
TDP43del274_320fw	This study	GCCATGATGGCTGCCGCCCA
TDP43del274_320rev	This study	ACTTCTTTCTAACTGTCTATTGCTATTGTGCTTAG
TDP43del367_414fw	This study	TAAACCCGCTGATCAgccTCGAC
TDP43del367_414rev	This study	GGCCTGGTTTGGCTCCCTCTG
hnRNPA2truncRv	This study	ACAGCGCTCGAGACTCCTAGAACTCTGA ACTTCCTGCAT
NLS-hnRNPA2fw	This study	acagcgAGATCTGATCCAAAAAAGAAGAG AAAGGTAGAGAAAACTTTAGAAACTGTT CCTTTGGAGAGGA
hybrid linker-TDP43LC-fw Xba1	This study	acagcgTCTAGACAAGAAATGCAGGAAGTT CAGTTAGAAAGAAGTGGAAGATTTGGTGGT
TDP43LC-rv Xho1	This study	acagcgCTCGAGCGGCCGCCACTG
NLS-pGEX-3x-hnRNPA2-fw	This study	acagcgAGATCTGATCCAAAAAAGAAGAGA AAGGTAGAGAGAGAAAAGGAACAGTTCC GTAAGCTCTTTA
pGEX-3x-hnRNPA2 fw	This study	acagcgAGATCTGAGAGAGAAAAGGAACAG TTCCGTAAGCTCTTTA
A2cMycNLS_ BsrGI_ pCDNA3_mCHerry_fw	This study	GACGAGCTGTACAAGcctgctgctaagagagt gaaactggatGAGAGAGAAAAGGAACAGTT CCGTAAG
A2trunc_EcoRI_ pCDNA3_mCHerry_rev	This study	CTGCAGAATTCTCAACTCCTAGAACTCT GAACTTCCTGC
A2TDPCTD_EcoRI_ pCDNA3_mCHerry_rev	This study	CTGCAGAATTCCTACATTCCCCAGCCA GAAGACT
TDP43fl_fw	This study	aaaaggatccATGgagcaaaagctcatttc
TDP43fl_rev	This study	aaaactcgaGCTACATTCCCCAGCCAGAAGACT
MH239_mutA326P_fw	This study	GATGGCTGCCCCCCAGGCAGCACTACA
MH240_mutA326P_rev	This study	TGTAGTGCTGCCTGGGGGGCAGCCATC
MH241_mutQ331K_fw	This study	AGGCAGCACTAAAGAGCAGTTGGGGTATGATG
MH242_mutQ331K_rev	This study	CATCATACCCCAACTGCTCTTTAGTGCTGCCT
MH243_mutG294A_fw	This study	GTAATAGCAGAGCGGGTGGAGCTGGTTTGGGAA
MH244_mutG294A_rev	This study	TTCCCAAACCAGCTCCACCCGCTCTGCTATTAC
MH251_mutG335A_fw	This study	TACAGAGCAGTTGGGCTATGATGGGCATG
MH252_mutG335A_rev	This study	CATGCCCATCATAGCCCAACTGCTCTGTA
MH253_mutM337P_fw	This study	CAGTTGGGGTATGCCGGGCATGTTAGC
MH254_mutM337P_rev	This study	GCTAACATGCCCGGCATACCCCAACTG
pJ4M_TDP-43 del272_320fw	This study	GCAATGATGGCGGCTGCACAA
pJ4M_TDP-43 del272_320rev	This study	GGAACGTTCCAGTTGGCGGT
pJ4M_TDP-43 316delUCR346fw	This study	TCAGGCCCGAGCGGCAATAATCAG
pJ4M_TDP-43 316delUCR346rev	This study	GGCACCAAAGTTCATACCGCCACCCA
pJ4M_TDP-43 320delQN366fw	This study	TTTGGTAGCGGTAACAATAGCTACAGCG
pJ4M_TDP-43 320delQN366rev	This study	CGGATTGATGGAGAAGGCACCAAAG
pJ4M_TDP-43 320del414rev	This study	CGGATTGATGGAGAAGGCACCAAAGTTCA
pJ4M_TDP-43 367del414rev	This study	CGCTTGATTCGGTTCACGCTGCATGT
SMC1A PAS distal_fw	(Rot et al., 2017)	caaccaaagaagtcacgtacca
SMC1A PAS distal_rev	(Rot et al., 2017)	aattgtgctcgtccataaagg
SMC1A PAS prox_fw	(Rot et al., 2017)	GTTCTACACCAAGGCCGAGA
SMC1A PAS prox_rev	(Rot et al., 2017)	TCGAAGGTCAGGACTTTGCT
PPP2R2D GE fw	This study	TTGAGTGTTGCTGGAACGGT
PPP2R2D GE rev	This study	TACACACCTTCCGGGGTTTG
PPP2R2D APA fw	This study	TGATTGCCTGTGCCCCTG
PPP2R2D APA rev	This study	TAGACAGGGGGATGGGATGG
GPCPD1 GE fw	This study	AGCAGGAATTGCCAGAGCTT
GPCPD1 GE rev	This study	ACTGAGAAGCCCAAAAGGCA
GPCPD1 dist fw	This study	GGAAAGTGTTGTGGCGCTTC
GPCPD1 dist rev	This study	TGGATGGGAGACGACAGACA
GXYLT1 GE fw	This study	TGACGATAAGCAACCAGCAT
GXYLT1 GE rev	This study	TGGTGATCTGGCATAACGATCT
GXYLT1 APA fw	This study	AGGGTCCCTGGTCAGACATT
GXYLT1 APA rev	This study	ACAAGAGGTTGCTATAGTGTGCT
ATG4B fw	(Ling et al., 2015)	TGTGTCTGGATGTGAGCGTG
ATG4B rev	(Ling et al., 2015)	TCTAGGGACAGGTTCAGGACG
GAPDH qPCR fw	This study	AATCCCATCACCATCTTCCAG
GAPDH qPCR rev	This study	AAATGAGCCCCAGCCTTC

**Recombinant DNA**		

pcDNA 3.1(+) Mammalian Expression Vector	Thermo Fisher Scientific	Cat#V79020
pOG44 Flp-Recombinase Expression Vector	Thermo Fisher Scientific	Cat#V600520
pcDNA5 FRT/TO Vector Kit	Thermo Fisher Scientific	Cat#V652020

**Software and algorithms**		

Fiji/ImageJ	Rueden et al., 2017	https://imagej.nih.gov/ij/
GraphPad Prism 5	GraphPad Software	https://www.graphpad.com/scientific-software/prism/
R v 4.0.3	The R Project for Statistical Computing	https://www.r-project.org/
Python v 3.7	Python Software Foundation	https://www.python.org
Snakemake v 5.31.1	Mölder et al., 2021	https://snakemake.github.io/
iCount; iMaps	König et al., 2010	https://github.com/tomazc/iCount
iCLIP analysis code; pAseq analysis pipeline and code	This study	https://github.com/ulelab/tdp43-mutants

### Resource availability

#### Lead contact

Further information and requests for resources and reagents should be directed to and will be fulfilled by the lead contact, Jernej Ule (jernej.ule@crick.ac.uk).

#### Materials availability

All unique/stable reagents generated in this study are available from the lead contact.

### Experimental model and subject details

#### *In vitro* phase separation assays

##### Purification of Recombinant TDP-43 MBP His6

Plasmids, pJ4M TDP-43 wild-type (WT) MBP His6 pJ4M/TDP-43 was as described (Addgene plasmid #104480; http://addgene.org/104480; RRID:Addgene_104480) (Wang et al., 2018). Deletions pJ4M TDP-43 272del320 MBP His6, pJ4M TDP-43 316del346 MBP His6, pJ4M TDP-43 321del366 MBP His6, pJ4M TDP-43 320del414 MBP His6 and pJ4M TDP-43 367del414 were generated by PCR with phosphorylated oligos (Key resources table) flanking the deleted regions and amplification of the entire plasmid. The resulting reactions were Dpn1 treated, ligated and transformed into DH5alpha. The whole locus was then sub-cloned into the original vector backbone to avoid unintended PCR-generated mutations.

All constructs were purified using two different methods. For proteins used for droplet formation using dextran, the plasmids were first transformed into BL21(DE3)RIL *E. coli*. A small culture of the cells was grown at 37°C in LB media containing antibiotics, kanamycin (50μg/mL) and chloramphenicol (34μg/mL). After approximately 4 hours, the cells were transferred to a larger culture with 0.2% (w/v) glucose and grown to OD600 of about 0.5. The protein expression was induced with 1mM isopropyl β D 1-thiogalactopyronoside (IPTG) and the cells were grown overnight in 15°C. The cells were harvested via centrifugation at 4,658 g for 20 minutes at 4°C and re-suspended in 20mM Tris-HCl (pH 8.0), 1M NaCl, 10mM imidazole, 10% glycerol and 1mM DTT with EDTA-free protease inhibitor (cOmplete, EDTA-free, Roche). The re-suspended cells were subsequently lysed using Misonix sonicator 3000 after incubation with lysozyme (20μg/mL) on ice for 30 minutes. The proteins were purified over Ni-NTA agarose beads (Thermo) and eluted in 20mM Tris-HCl (pH 8.0), 1M NaCl, 300mM imidazole, 10% glycerol and 1mM DTT. The eluate was further purified over amylose resin (NEB) and eluted in 20mM Tris-HCl (pH 8.0), 1M NaCl, 10mM imidazole, 10% glycerol, 10mM maltose and 1mM DTT. The eluted protein was concentrated to approximately 150μM using Amicon® Ultra Centrifugal filters (50K, Millipore) and stored in aliquots in −80°C after being flash-frozen in liquid N2. Molecular weights were determined by polyacrylamide gel electrophoresis using 4%–20% Tris-HCl gel (BioRad) followed by staining with Coomassie Brilliant Blue (BioRad).

For droplet formation using TEV protease, all constructs were purified as described in Wang et al. (2018). Briefly, plasmids were transformed into BL21 Star (DE3) *E. coli*. Cell cultures were grown at 37°C to an OD600 of 0.6-0.9, then cooled down to 16°C. Protein expression was induced overnight with 1mM IPTG at 16°C. Cells were harvested by centrifugation and resuspended in 20mM Tris-HCl (pH 8.0), 1 M NaCl, 10 mM imidazole, 10% glycerol, 1 mM DTT supplemented with EDTA-free protease inhibitor cocktail tablets (cOmplete, EDTA-free, Roche). Cells were lysed with Emulsiflex C3 (four passes), and clarified by centrifugation at 48,000 g for 1 hour. Clarified lysates were filtered using 0.2 μm syringe filter and applied to a 5 mL HisTrap HP column. Proteins were eluted with a linear gradient of elution buffer (20mM Tris-HCl (pH 8.0), 1 M NaCl, 500 mM imidazole, 10% glycerol, 1 mM DTT). Eluant was further purified over HiLoad 26/600 Superdex 200 pg (GE Healthcare) with 20 mM Tris-HCl (pH 8.0), 300 mM NaCl, and 1 mM DTT. As described in Wang et al., 2018, size exclusion results in three distinct eluant peaks in the following order of retention time: TDP-43-MBP:nucleic acid aggregates (peak 1), pure TDP-43-MBP oligomers and monomers (peak 2), and contaminants (peak 3). For each TDP-43 variant, the second peak was pooled and concentrated to approximately 240 μM using Amicon® Ultra Centrifugal filters (50K, Millipore), flash frozen, and stored at −80°C.

##### Total RNA extraction from HeLa cells

HeLa cells were cultured to 90% confluence in DMEM media supplemented with 10% FBS and penicillin/streptomycin at 37°C at 5% CO_2_. Media was aspirated and cells were lysed in 1ml TRIzol reagent (Invitrogen, catalog number 15596206) per 10cm culture dish, homogenized by pipetting, transferred to a DNA/RNA low-bind tube and incubated for 5 mins at room temperature. 0.2ml chloroform/1ml TRIzol reagent was added and the sample was centrifuged at 12,000 x g for 15 minutes at 4°C. 500μl of isopropanol was added per 1/ml TRIzol, incubated at room temperature for 10 minutes, and the sample was centrifuged for at 12,000 x g for 10 minutes at 4°C. The pellet was dislodged and washed in 1ml 75% ethanol and centrifuged at 7,500 x g for 5 minutes at 4°C. The RNA was resuspended in 50μl of RNase free water per 10cm culture for use in TDP-43 droplet formation experiments.

##### Droplet formation

Droplet formation using dextran: WT TDP-43-MBP and the deletion constructs were thawed on ice and centrifuged for 10 minutes at 16,100 g at 4°C. The proteins were subsequently buffer exchanged into 20mM HEPES-NaOH (pH 7.4), 150mM NaCl and 1mM DTT using Micro Bio-Spin P-6 (BioRad). Corresponding extinction coefficients and molecular weights of the proteins were determined using ExPASy ProtParam software and were used to quantify the protein concentrations by NanoDrop (ThermoFisher). The phase separation reaction was set up with 10% (w/v) Dextran, 20mM HEPES-NaOH (pH 7.4), 150mM NaCl and 1mM DTT. WT and deletions of TDP-43 were added last to the reaction to final concentrations of 10μM. Phase-separated droplets were imaged by DIC microscopy after 30 minutes of incubation in room temperature. Droplets smaller than 2.5μm in diameter, sparsely distributed across the image were categorized as “sparse, small droplets.” Droplets greater than 2.5μm in diameter and densely packed were categorized as “droplets.” For the turbidity measurements, absorbance values of phase-separated proteins were read at 395nm using TECAN (Safire^2^).

Droplet formation using TEV protease: WT TDP-43-MBP and deletion constructs were thawed on ice and centrifuged for 10 minutes at 16,100 g. Protein concentrations were then determined by NanoDrop, as described above. Proteins were diluted to 0.1-10 μM in 20mM HEPES-NaOH (pH 7.4), 150mM NaCl and 1mM DTT. Droplet formation was initiated by the addition of TEV protease at a final concentration of 0.03 mg/mL. In some experiments, total HeLa cell RNA was included at 5-40ng/μl. After 30 minutes of incubation, phase-separated droplets were imaged by Brightfield microscopy (EVOS M5000). Turbidity measurements were recorded as described above.

##### Saturation concentration (Csat) determination by centrifugation

To determine C_sat_ of WT TDP-43-MBP and the deletions, phase-separated reactions with 10μM proteins in 10% (w/v) Dextran, 20mM HEPES-NaOH (pH7.4), 150mM NaCl and 1mM DTT (see Droplet formation for detailed protocol) were centrifuged for 30 minutes at 21,130 g in room temperature. 2μL of the supernatant was sampled carefully without disturbing the pellet and the concentration was quantified using the NanoDrop (see Droplet formation for more information).

##### 1,6-HD treatment of preformed TDP-43-MBP condensates

Purified TDP-43-MBP was buffer exchanged into LLPS buffer (150 mM NaCl, 20mM HEPES, 1mM DTT) with Micro Bio-Spin P-6 columns (Biorad). Preformed TDP43-MBP droplets were prepared with 5.7μM TDP-43, 8.6% dextran sulfate in buffer and incubated at room temperature for 15 minutes before adding either buffer or 1,6-HD for final concentrations of 5μM TDP-43, 7.5% dextran sulfate, 8% 1,6-HD (w/v) in LLPS buffer. Sample absorbance was measured by Tecan Safire^2^ (395 nm, 10 reads/measurement) 20 minutes after adding buffer or 1,6-HD. Absorbance readings from blank samples (no TDP-43 added) were subtracted from sample readings. For imaging, TDP-43-MBP droplets were prepared as described, with the addition of Alexa488-labeled TDP-43-MBP (Alexa Flour™ 488 NHS Ester, 1:200 labeled:unlabeled). TDP-43-MBP droplets were imaged 10-30 minutes after addition of buffer or 1,6-HD with 60x objective on EVOS M5000. Droplet size and area fraction were quantified using Cell Profiler (Version, 4.0.7, identify primary objects, min diameter = 3 pixels, Robust Background, lower outlier fraction = 0.05, threshold smoothing scale = 1.3488).

### Method details

#### Mammalian cell culture

HEK293 Flp-In T-REx cells were maintained in Dulbecco’s Modified Eagle Medium (DMEM) with 10% fetal bovine serum (FBS), supplemented with 3 μg/ml blasticidine and 50 μg/ml zeocin. HEK Flp-In T-Rex cell lines were generated by co-transfection of pcDNA5/FRT/TO/GFP constructs together with pOG44 (Invitrogen). Stable integrates were selected by culturing cells in DMEM with 250 μg/ml hygromycin, 4 μg/ml blasticidine.

#### Generation of GFP TDP-43 inducible cell lines

Full-length constructs and mutants of TDP-43 tagged with GFP were inserted into the pcDNA5/FRT/TO plasmid (Life Technologies, V6520-20). In order to generate siRNA resistance in TDP-43, a region of the TDP-43 coding sequence (5′-GAGCCAATTGAAATCCCAAGCGAA-3′) was silently mutated. Point mutations were generated by site-directed mutagenesis following Quikchange Site-directed mutagenesis instructions (Agilent Technologies) using oligonucleotides detailed in Key resources table. All primers and oligonucleotides for this study were ordered from IDT (Integrated DNA Technologies). Deletions were generated by PCR with phosphorylated oligos (Key resources table) flanking the deleted regions and amplification of the entire plasmid. The resulting reactions were Dpn1 treated, ligated and transformed into DH5alpha. The whole locus was then sub-cloned into the original vector backbone to avoid unintended PCR-generated mutations.

Full-length and deletion constructs of hnRNPA2 (cDNA amplified with primers in Key resources table) tagged with GFP were inserted into the pcDNA5/FRT/TO plasmid (Life Technologies, V6520-20). For truncation and the chimeric A2-TARDBP-IDR an N-terminal addition of an SV40 NLS was required to compensate for loss of C-terminal non-canonical NLS. To create the chimeric gene a hybrid linker - partly hnRNPA2, partly TARDBP CTD - was used to clone the IDR via an Xba1 site to the RRMs of hnRNPA2. Stable Flp-In lines were produced as above.

For the co-expression experiments hnRNPA2 constructs were tagged with the mCherry fluorescent protein. Therefore the hnRNPA2_TDP_CTD and hnRNPA2_delCTD constructs were amplified from the pcDNA5/FRT/TO plasmids with primers (Key resources table) containing BsrGI and a cMYC NLS and a EcoRI-containing primer into pcDNA3.1 N-mCherry vector.

#### Microscopy and imaging analysis

##### Z stack confocal imaging

Glass coverslips were pre-coated with poly-D-lysine (0.5mg/ml) for at least 1 hour, then washed with distilled water (cell-culture grade, GIBCO 15230188) twice and air-dried in the hood for half an hour. GFP-TDP-43 cells or GFP-hnRNPA2 cells were seeded on pre-coated glass coverslips. After 24 hours, doxycycline (150ng/ml) was added to the media to induce GFP-TDP-43/hnRNPA2 expression for 24 hours unless specified otherwise for the time course experiment (4-72 hours). For coexpression imaging experiments between mCherry-hnRNPA2 and GFP-TDP-43, GFP-TDP-43-WT Flp-IN cells seeded on pre-coated glass coverslips in 6 well plates were transfected with mCherry-hnRNPA2 plasmids (or no plasmid control) using lipofectamine 3000 following manufacturer’s protocol at 1.25ug per 6 well. After 4 hours, media was exchanged and doxycycline was added to induce GFP-TDP-43 WT expression for 24 hours. Cells were fixed in 4% PFA in 1xPBS for 15min, and washed 3x PBS. Coverslips were then mounted with VECTASHIELD (Vector labs, H-1000).

0.35 μm z stacks of HEK293 Flp-In cell lines were obtained with Zeiss inverted 880 confocal microscope with a 63x objective at 4x zoom. Dimensions were set at 2048x2048 pixels. Pinhole size was set to 1 airy unit.

##### Quantification of fluorescence intensity from confocal z stacks and counting procedure

Quantification of fluorescence intensity and GFP-TDP-43 foci counting were done using a custom-written macro for batch processing in Fiji, based on the procedure from Jain and Vale (2017). For each z stack, the maximum intensity z-projection was used for automatic segmentation of the nuclei signal using the GFP-signal, taking advantage of the observation that GFP-TDP-43 signal was predominantly in the nucleus (hence no independent nuclei staining such as DAPI was required). Maximum intensity z-projection was first smoothed with Gaussian and Median filtering, then auto-thresholded with the default settings in Fiji. The Adjustable Watershed plugin was set with a tolerance = 40, to separate nuclei which were touching each other. Masks were created for each segmented nuclei from each image, and measurements of mean/median, skewness, kurtosis, standard deviation of the fluorescence intensities and the area were taken from each mask. To filter out measurements from masks containing more than one nuclei, all masks with an area > 300 pixels and the associated measurements were discarded.

The foci counting and segmentation procedure was automated in order to eliminate variability associated with manual counting of ‘foci’. Consistency of manual counting would be particularly difficult in this case especially across nuclei of different fluorescence intensities. To count the number of nuclear GFP-TDP-43 foci for each cell line, the masks associated with each nuclei created from the maximum intensity projection were overlaid and used to segment the original z stack. Subsequently nuclear GFP foci from the segmented z- stack for each nuclei was counted with the ImageJ 3D Objects Counter (Bolte and Cordelières, 2006). A relative intensity threshold was set as 1.6x of the mean fluorescence intensity (Jain and Vale, 2017) of the z-projection to account for variability of GFP-TDP-43 expression across cell lines and individual cells. To quantify the area and mean fluorescence intensity of the condensed fraction, foci ROI from ‘Object map’ output from ImageJ 3D Objects Counter was overlapped with the maximum intensity z-projection of each nucleus.

To compare GFP-TDP-43 foci counts in co-expression imaging experiments between mCherry-hnRNPA2 and GFP-TDP-43 from the transfected coverslips, nuclear segmentation and foci counting were carried out as before with the GFP channel. Separately, the derived nuclear masks were used to segment the mCherry channel of the maximum z-projection. Each field of view contained cells expressing heterogenous levels of mCherry-hnRNPA2. Image files of the mCherry channel of each segmented nucleus were anonymized and shuffled for blinded manual classification into ‘low’ (no or low expression) or ‘high’ (medium or high levels) hnRNPA2 expression based on visualizing the mCherry signal using the ImageJ ‘Blind Analysis Tools’ plugin ‘Analysis and Decide’ function. Blinded classification results were then subsequently matched to the GFP foci counts.

#### Fluorescence recovery after photobleaching (FRAP)

GFP-TDP-43 HEK293 Flp-In cells were seeded 24 to 48 hours prior to the experiment on Glass-bottom cell culture dishes (Nunc 150680), pre-coated with poly-D-lysine, as before. Doxycycline was added to the media 24 hours prior to the experiment to induce GFP-TDP-43 expression at a near endogenous level. DMEM 10% FBS media was replaced with Fluorobrite DMEM (GIBCO, A1896701), 2% FBS + Glutamax supplemented with doxycycline, 1-2 hours prior to the start of FRAP experiments. FRAP was carried out on Zeiss inverted 880 confocal microscope, equipped with a 63x objective, a gas mixer CO_2_ supply and a temperature-controlled chamber set at 37°C, with Zeiss 2012 software. All parameters were kept constant across independent experiments and conditions.

Acquisition parameters: EGFP channel, Frame size: 512x512, 12 bit, Averaging = 1, Bidirectional scanning, scan speed = 9, Scan area = 4x during FRAP acquisition, Pinhole: 90.1 μm, Gain: 750, Bleaching parameters (This should reach > 95% bleaching efficiency on fixed cells): 100% laser power on 488nm laser, 20 bleach iterations, Start bleach after 5 cycles, Different scan speed for bleaching: 6, ZOOM bleach, Bleach area = rectangle of 20x24 pixels at 4x zoom, Acquisition cycles: 200 cycles, frame interval = 326ms. Hence fluorescence recovery is monitored for around 1 minute, where plateau is reached. The bleaching area was a small nuclear region outside of any foci (Figure S2D), unless otherwise specified. For foci-centered bleaching, the bleaching area was centered on the selected foci within the nucleus (Figure S3C).

#### FRAP analysis

FRAP analysis was done with the FRAP calculator package (by Robert Bagnell; https://www.med.unc.edu/microscopy) on ImageJ (Rueden et al., 2017). FRAP series were imported into ImageJ with the area of bleaching defined as a ‘region of interest’ (ROI). The frame interval was defined as 326ms and the plugin measured the mean pixel intensities across the entire acquisition cycles of the bleached area during the recovery phase. A control ROI of the same area in another nuclei in the same image was used to normalize for the pixel intensities to account for fluctuations in laser power and photobleaching of GFP signal during the entire acquisition series.

Since bleaching occurs at time point = 6, the fluorescence intensities at the first 5 pre-bleach time points were averaged and used again to normalize the fluorescence intensity values at all subsequent post-bleach time points. Fluorescence recovery plateaued within the 1 minute acquisition time. For the fluorescence recovery time course of each individual cell, fluorescence intensities at post-bleach time points up to 60 s were used to plot the FRAP curve and for non-linear regression (done in Prism), with the following settings: one phase association (i.e., single exponential), variance weighted by 1/Y^2^. This derives an approximate rate constant of FRAP. The mobile fraction was calculated from the plateau and the first post-bleach fluorescence intensity of the fitted curve with the following equation:

$$\left(Intensity_{\text{plateau}}-Intensity_{\text{initial post}-\text{bleach}}\right)\;/\;\left(1\;-Intensity_{\text{initial post}-\text{bleach}}\right)$$

#### Comparison of *in vitro* Csat with FRAP and foci measurements

Best fit parameters for sigmoidal fitting of Log(Csat) versus foci count were determined in Prism7 with the four-parameter logistic curve model:

$$Y=minimum+\left(maximum-minimum\right)/(1+10^{\wedge }\left(\left(Log\left(inflection\;point\right)-X)*HillSlope\right)\right)$$

Where maximum = 33.29, minimum = 2.215, inflection point = 6.30283109, HillSlope = −9.441. Best fit parameters for linear regression of Log(Csat) versus rate constant were determined in Prism7.

#### Statistics for image quantification

Quantification of FRAP (i.e., rate constant of FRAP and the mobile fraction of GFP-TDP-43 cell lines) and data derived from confocal z stacks were statistically tested for normality (Prism 5, Kolmogorov-Smirnov test, D’Agostino and Pearson omnibus normality test and Shapiro-Wilk normality test). Since there were conditions where the datasets did not pass a normality test, significance was tested with Kruskal-Wallis test followed by Dunn’s post hoc test for multiple comparisons. The p values reported were from the individual comparisons in Dunn’s test. In experiments comparing differences between cell lines plus an additional factor (si-TDP-43 or foci-centered), a two-way ANOVA was performed in Prism. The number of cells quantified for each condition is shown in the corresponding figure legends.

#### Individual-nucleotide resolution UV-crosslinking and immunoprecipitation of protein-RNA complexes (iCLIP)

This experiment identified TDP-43-RNA binding sites in 293 Hek Flip-In cells. Cells were grown to 80% confluence, transgene was induced by doxycycline for 24h, UV crosslinked on ice and then lysed in RIPA buffer. 0.4 Units of RNaseI (0.2 Units for Figure S5C) and 4 Units Turbo DNase were added per 1 mL of cell lysate at 1mg/ml protein concentration for RNA fragmentation. Negative controls (no-UV) were prepared. Antibodies against GFP coupled to magnetic Protein G beads or GFP-TRAP_MA beads (Chromotek, gtma-20) were used to isolate Protein-RNA complexes, and RNA was ligated to a pre-adenylated infra-red labeled IRL3 adaptor (Zarnegar et al., 2016) with the following sequence:

/5rApp/AG ATC GGA AGA GCG GTT CAG AAA AAA AAA AAA /iAzideN/AA AAA AAA AAA A/3Bio/

For experiment Figure S5B we chose to multiplex a maximum of 3 samples before the protein gel which required for the ligation step a bar-coded unlabeled L3 adaptor of the following design:

/5rApp/WN XXX AGA TCG GAA GAG CGG TTC AG/3Bio/

The complexes were then size-separated by SDS-PAGE, blotted onto nitrocellulose and visualized by Odyssey scanning. For the multiplexed sample, one replicate was run in parallel with the IRL3 to allow quality control of the RNP complexes on the membrane and to help with cutting of the bands. RNA was released from the membrane by proteinase K digestion and recovered by precipitation. cDNA was synthesized with Superscript IV Reverse Transcriptase (Life Technologies) and AMPure XP beads purification (Beckman-Coulter, USA), then circularized using Circligase II (Epicenter) followed by AMPure XP beads purification. After PCR amplification, libraries were size-selected with Ampure beads (if necessary by gel-purification) and quality controlled for sequencing. Libraries were sequenced as single end 100bp reads on Illumina HiSeq 4000.

For the ‘RNase experiment’ we use 4 units Turbo DNase and 0.1 (low), 0.4 (medium) or 2 units (high) per 1 mL of lysate at 1mg/ml protein concentration. This dataset was used for the metaprofile of motif coverage in Figures 4C–4E. For the ‘chimeraRBP-CLIP’ GFP-TRAP_MA beads (Chromotek, gtma-20) were used for induced GFP-tagged protein variants, and for endogenous protein, Protein G Dynabeads (Life Technologies) were coupled to Antibodies against hnRNPA2B1 (SC374052, Santa Cruz). For the ‘HD dataset’, cells were treated with 8% 1,6-Hexanediol (HD) or 2.5-HD (for Figure S5D) in DMEM for 5 min at 37 degrees, and then cells were washed in ice-cold PBS, cross-linked and library preparation was performed as described above.

#### Western blot analysis

For the TDP-43 autoregulation experiment, the cell lines were induced with 150 ng/ml doxycycline (Dox) for 48 h. In parallel a set of uninduced samples was processed. Cells were harvested, washed once with PBS and lysed with 300 μL of RIPA lysis buffer (25 mM Tris-HCl pH 7.6, 150 mM NaCl, 1% NP-40, 1% sodium deoxycholate, 0.1% SDS) supplemented with 25x Protease Inhibitor Cocktail (Roche) and Benzonase Nuclease (Novagen) for 5 min at 4°C. Supernatants were cleared of debris by 10 min centrifugation at 13000 rpm at 4 °C and total protein concentrations were determined using the DC Protein Assay Kit (Bio-Rad). 2 μg of total cell lysates were supplemented with 4x Nupage loading buffer (+DTT, fc. 10mM) and separated over 4%–12% gradient SDS-PAGE gels, transferred to a 0.2 μm nitrocellulose membrane using the Trans-Blot Turbo RTA Mini Nitrocellulose Transfer Kit (Bio-Rad) and blotted with rabbit polyclonal anti-TDP-43 (10782-2-AP Proteintech), and mouse monoclonal anti-α-Tubulin (T5168 Sigma-Aldrich). After secondary antibody incubations, signals were detected by LI-COR secondary Antibodies (IRdye680 1:15000, IRdye800 1:15000) and visualized by Odyssey scanning (LI-COR) and quantified using Image Studio Lite (LI-COR).

#### siRNA transfection

For the siRNA-induced knockdown of TDP-43, 240 pmol of TDP-43 stealth siRNA was mixed with 10 μL of RNAiMAX following the manufacturer’s reverse transfection protocol and added to a 10 cm dish of HEK293 Flp-In cells. After the first 24hrs of transfection, the medium was replaced with DMEM with 10% FBS and after an additional 24hrs, the cells were collected for analysis. The scrambled control siRNA was used at 240 pmol to distinguish off-target effects from biologically relevant ones. For rescue experiments with stable cell lines, 24hrs after the transfection, the medium was replaced with DMEM with 10% FBS and 150 ng/mL doxycycline for the induction of the protein of interest, and cells were collected 24h after induction. For 6-well dishes the reactions were scaled down accordingly.

#### Preparation of total RNA

Cells were washed once with PBS and harvested by centrifugation. RNA was extracted from cell pellets using Maxwell RSC simply RNA cells kit in the Maxwell RSC instrument following manufacturer’s instructions.

#### RT-PCR

The Superscript II was used for the reverse transcription reaction with oligo-dT according to manufacturer’s instructions (Thermo Scientific). Between 500-1000 ng RNA was used as input. For PAS switches we used qPCR, whereby 2 μL of diluted cDNA was used for each reaction using SYBR green PCR mastermix and each primer at a final concentration of 0.2uM in a QuantStudio 6 Flex Real-Time PCR System. To analyze the PAS changes, relative expression values were normalized against the gene expression values of primers in the gene body. In the case of ATG4B cryptic exon inclusion levels were normalized against GAPDH expression. Reactions were carried out on biological triplicates and technical duplicates. Oligos used for qPCR are listed in Key resources table.

#### Generation of pAseq libraries

To quantify poly(A) site usage, we used a customized Quantseq 3′ end sequencing method that allowed us to multiplex cDNAs straight after the reverse transcription with a barcoded RT-primer (barcode position as xxxxx in RT-primer below). The samples were pooled into subgroups of 6 and each group had a separate Lexogen barcode (i7 indices). The libraries were sequenced with 100-nt paired-end reads on HiSeq, such that the experimental barcode of the RT primer was acquired in read 2, and the Lexogen barcode by the index read.

For most of the protocol, we used the forward QuantSeq mRNA 3′ end sequencing kit (Lexogen) according to manufacturer’s recommendations. Libraries were prepared from cells siRNA-depleted of TDP-43 with and without rescue with the transgene induced for 24h by Dox treatment or with transgene induction only. Also 1 replicate of each cell line without any treatment was analyzed to monitor cell-line specific variations in gene expression. We modified the standard protocol to enable multiplexing of 6 individual libraries straight after RT:

5 μL of RNA (100ng – 1ug) were mixed with 5 μL of custom RT primer (12.5 nM final concentration) and 5 μL of FS1x. FS1x and FS2x come in QuantSeq-Flex Targeted RNA-Seq Library Prep Kit V2.

3′Seq_RT 5′BioGTTCAGACGTGTGCTCTTCCGATCTxxxxx TTTTTTTTTTTTTTTTTTTTVN-3′

Samples were denatured for 3 minutes at 85 degrees then cooled to 42°C.

FS2x/E1 mastermix (pre-warmed to 42°C) was added and kept in the thermocycler at 42°C to reduce internal mispriming. RT was run for 15 minutes at 42°C after which the Reverse Transcriptase was inactivated at 70°C for 10 minutes. Inactivation of the RT helps prevent any cross/mis-hybridization effects after pooling.

Up to 6 samples were pooled and after pooling were immediately purified to remove buffers, excess RT primer, enzyme and to reduce the volume. 120 μL of pooled sample was purified with 2.5x Purification Beads (supplied with QuantSeq Flex kit); cDNA was eluted in 50 μL of water. 10.5 μL of this pool was used in second strand synthesis by adding 5 μL FS1x, 4.5 μL FS2x and 5 μL RS. RNA was removed by heat treatment at 95°C for 10 minutes. Second strand synthesis reactions for each pool were conducted according to the standard Quantseq protocol. All libraries were sequenced on Illumina HiSeq 4000 machines in a paired-end manner with a read length of 100 nt, and an additional 10nt index read. We used multiple replicates per cell line in two experimental batches (knockdown v. rescue - 316del346: 9 v. 9; A326P: 8 v. 9; M337P: 9 v. 8; Q331K: 3 v. 3; G294A: 3 v. 2; G335A: 8 v. 9; WT: 8 v. 9).

#### Computational analyses

##### Conservation and Disorder score calculation

Amino acid conservation of TDP-43 C-terminal domain was calculated using https://consurf.tau.ac.il, applying default settings. The disorder confidence score is shown for the C-terminal domain, calculated using the DISOPRED3 algorithm with default settings on the full length TDP-43 amino acid sequence on the PSIPRED server (http://bioinf.cs.ucl.ac.uk/psipre).

##### Analysis of iCLIP data

Reads were processed according to standardized iCLIP analysis methods using the iMaps webserver, and are available at processed on the iMaps webserver:

https://imaps.goodwright.org/collections/868/

For all samples the human GRCh38 genome build and GENCODE version 27 annotation was used. Customized downstream analysis of iCLIP data was done using scripts described below, and written in Python 3.7.3.

##### Regional thresholding to obtain thresholded crosslinks (tXn)

Thresholded crosslinks (tXn) were identified as the starting point for the scripts that are described further below. Crosslinks with high cDNA counts were identified based on thresholds that were dynamically calculated for each transcript region. Each intron, each intergenic region, and combined exons in each gene were defined as their own regions. Intervals with a high density of crosslinks and a high cDNA counts per crosslink site (peaks) were called using Paraclu (Frith et al., 2008) with parameters: minValue of 10 and max cluster length 200 (default). Crosslink sites in each region that have a cDNA count equal or above the 70th percentile (or as described below) of counts of crosslinks that are in the peaks in the region were then defined as ‘thresholded crosslinks’ (tXn).

##### Kmer analysis with positionally enriched kmer analysis (PEKA)

PEKA was used to identify the 6mers that were most enriched around crosslinks in the iCLIP data. In this study we used PEKA with introns, because they contained the vast majority of crosslinks (Figure S4B), and we find that nuclear RBPs generally have the strongest specificity at intronic crosslink sites. We aimed to identify motifs that enable high-affinity binding of the corresponding protein while avoiding identification of crosslinking preferences (commonly U-rich sequences; Haberman et al., 2017; Sugimoto et al., 2015) or other experimental artifacts common to all crosslink sites. To do so we identified kmers that are enriched at specific positions around tXn as compared to remaining crosslinks that are present outside peaks (oXn) (which more likely represent weak binding sites). The genomic sequences flanking the crosslink sites were separated into proximal and distal regions. The proximal region was −40 to 40nt relative to each crosslink site, and the distal regions were −150 to −100nt and 100 to 150nt relative to each crosslink site. For each motif, the positions of enrichment were identified in the proximal region by analyzing their normalized occurrence around all tXn within the examined region, which was obtained by dividing the occurrence at each proximal position by the average occurrence across all distal positions. Thus, PEKA examined the enrichment of each motif in the context of its regional composition. Positions chosen for further analysis included all positions in the region −13..13nt relative to the crosslink site, as well as those in the regions −40..-14 and 14..40nt where the normalized occurrence was greater than 2. An average normalized enrichment PEKA score was calculated for each kmer across all chosen positions for tXn by comparing with the same positions around 100 control groups of crosslink sites that were randomly sampled from the oXn. The kmers were then ranked by PEKA score, and the top ranking motifs were selected for visualizing their coverage around crosslinks.

##### Comparative PEKA

Comparative PEKA was used to identify the 6mers that are most enriched in the iCLIP data of the TDP-43 variants (using all data from the ‘Mutants, low RNase’ experiment, (Table S1) by comparing iCLIP of the GFP-TDP-43 mutants or HD treated cells with the reference untreated wild-type GFP-TDP-43). The analysis was done with two sequential runs of PEKA. First, we analyzed the WT data with PEKA, using the merged iCLIP replicates of WT transgene (WT_1 and WT_2 from the ‘Mutants, low RNase’ experiment) and focusing on intronic regions. The first iteration of PEKA was run as described above, except that the chosen positions for further analysis include only those within −40..40 of tXn with the normalized occurrence greater than 4 (the higher threshold was possible due the high quality of the present iCLIP data, and it limited the motif search to the most relevant positions). This generated multiple parameters that were then used for a 2nd iteration of PEKA: the average distal occurrences around tXn for each kmer, which was used for data normalization; the positions relative to tXn that were used for enrichment calculations of all motifs; and all parameters for remaining crosslinks sampled from the referenced sample. In the 2nd iteration of PEKA, these pre-defined parameters were used to analyze the intronic regions of all the samples from the ‘mutants’ experiment. In this 2nd iteration, only the occurrence of the kmer at each specific position relative to tXn was calculated for each sample, whereas all other parameters were used from the 1st iteration. This ensured that the motif analyses were normalized in the same way and used the same relative positions for all case samples, thus enabling the comparison of motif enrichment to reflect purely the differences in motif occurrence around tXn between samples.

The 6mers were then ranked by their average PEKA score across all TDP-43 variants, and the 20 top ranked 6mers were used for visualization on a heatmap (Figure 4B), where motif log_2_(enrichment) values were averaged between replicate experiments, and normalized by subtracting the average log_2_(enrichment) across variants in order to visualize the relative enrichment of each motif across the TDP-43 variants. In the heatmap, the 20 motifs were then sorted based on their gradient of relative enrichment in the WT and condensation-promoting G335A variant compared with the condensation-deficient A326P and 316del346 variants, which was the basis of their subdivision into three groups. The first group had the clearest decrease in normalized iCLIP counts of the condensation-deficient variants (by a factor of > 1.3), the second group had modest decrease (by a factor of 1.1-1.3), and the third group did not have any decrease. Motifs from each group most often had characteristic divergence from UG-repeat ([UG]n), therefore they are referred to in the text and figures as YG-containing [UG]n, YA-containing [UG]n and AA–containing [UG]n respectively.

##### Metaprofile of average motif coverage around crosslinks

We visualized the coverage of groups of 6mers around the crosslink events in introns (Figures 4C–4E and 4G–4J) and 3′ UTRs (Figures S4C and S4F). Sequences flanking the crosslinks were scanned with a rolling window equal to the motif length. The cDNA count of crosslink positions that were identified by more than 20 unique cDNAs was capped at 20. All positions containing a motif were given a score corresponding to the cDNA count of the evaluated crosslink position and remaining positions were scored 0. Scores at each position around crosslinks in the assessed region were summed and divided by the total cDNA count of all evaluated crosslinks to generate the coverage showing the percent crosslink events overlapping with any 6-mer from the group at each position. Finally, coverage distributions were converted into smoothed lines using rollmean function with window size of 6.

##### Motif- and iCLIP-based binding region assignment

We identified the candidate binding sites of TDP-43 by looking for motifs that were located in the 60nt windows centered on each tXn. The 150 top-ranking 6mers identified by the comparative PEKA procedure were used and, as before, these were divided into three groups based on their relative enrichment across condensation-deficient versus condensation-capable variants (motifs from each group most often had characteristic divergence from UG-repeat, therefore they are referred to in the text and figures as YG-containing [UG]n, YA-containing [UG]n and AA-containing [UG]n respectively). tXn were defined based on combined data across ‘mutants’ and ‘HD’ experiments (which have the largest numbers of cDNAs, see Table S1) using the 50th percentile threshold. For each motif, the generic reference positions were identified based on summarized distribution around all examined tXn from intronic WT_2 data of the ‘mutants’ experiment, where the motif was enriched by a factor of > 4. We then proceeded to identify the binding sites based on motifs located around crosslink sites. We extracted sequences in a −30 to 30 nt window around these tXn, and scanned them for presence of queried motifs at the reference positions. This resulted in the ‘motif positions’ file for each of the three sets of motifs. The three sets of motif positions were combined and motif positions that were present within 30nt were merged into ‘binding regions’. Each of these regions was then allocated into one of 36 groups based on their length, motif density and the predominant type of [UG]n motifs that were present in the region (Figure 5) as follows:

First the regions were divided into 4 groups of following length range thresholds: 1-30nt, 31-60nt, 61-100nt, > 100nt. These boundaries were defined so that ± 40000 regions fell into each of the 1-30, 31-60, 61-100 class, while the > 100 class had less regions because the cDNA counts in this class were generally much higher, and thereby we ensured that the total count of cDNAs falling into each binding region class was comparable (Figure S5B).

Each of the resulting 4 groups were further subdivided based on the density of motifs (i.e., percentage of each binding region that was covered by motifs) into three classes of regions with relatively low, medium and high motif density, each containing an equal number of binding regions.

Each of the resulting 12 groups were then subdivided into further 3 classes each in the following order:

• Since the YG-containing [UG]n and AA-containing [UG]n motifs had the opposing trend in condensation sensitivity (Figure 4B), we first collected the 3000 top ranking regions (or top 1500 for the > 100 group) based on highest calculated ratio of YG-/AA-containing [UG]n motifs
• To obtain binding regions on the opposite spectrum of condensation sensitivity that were enriched in the insensitive motifs (AA-containing [UG]n), we then collected from remaining regions the 3000 top ranking regions (or top 1500 for > 100 group) based on highest calculated ratio of AA-containing [UG]n/remaining motifs
• All remaining regions are the third group

To study how the crosslinking behavior of the TDP-43 variants changed in each binding region class, we identified the cDNA/crosslink count for each region for each variant. For analyses presented in Figure 5, we included those regions where the total cDNA count in the ‘HD’ experiment (Table S1) was > 100, and those regions inside genes that had a cDNA count > 10 and contained at least 10% of the counts of the region that had the maximum count within the gene. For the visualization of individual motifs on specific binding regions (Figures 6 and 7), we only retained positions that overlapped with these filtered binding regions.

##### Visualization of iCLIP data

For the comparative visualization of iCLIP data, we normalized iCLIP cDNA/crosslink counts at a given crosslink site by the experimental library size. Normalized counts were then smoothed over the region of interest using a rolling mean, with a sliding window of 20 nt. The smoothed, normalized values were plotted across the region.

#### Analysis of pAseq data

##### Gene expression analysis

To quantify gene expression using the pAseq data we trimmed Illumina adapters, low-quality positions (Phred score < 10) and polyA tails using Cutadapt (Martin, 2011). We quantified gene counts using the pseudoaligner Salmon (Patro et al., 2017) and a decoy-aware transcriptome (created from the human genome, GRCh38, and the Gencode V33 annotation) specifying no read length correction as recommended for Quantseq. Salmon quantification output files were imported into R using tximport and counts normalized for visualization using the variance stabilizing transformation from DESeq2 (Love et al., 2014).

##### PolyA site usage analysis

The quality of the sequenced reads were checked with FastQC and Illumina adapters and low quality positions (Phred score < 10) trimmed using Cutadapt. First, we generated an atlas of PAS containing all detectable sites across all experiments. Reads that extended into the polyA tail were selected based on the presence of at least 5 consecutive ‘A’ bases at the 3′ end of the read using Cutadapt. These were then aligned against the human genome (GRCh38, with the Gencode V33 annotation) using STAR (Dobin et al., 2013). All alignment BAM files across all experiments were merged and the total genomic coverage of the 3′ ends of the reads calculated. Adjacent positions were merged into clusters and those with fewer than 2 reads were filtered. To assess for internal priming artifacts and filter them computationally, we used an approach similar to that described in Herzog et al. (2017). The percentage of A nucleotides in the 20 nucleotides downstream of each candidate PAS cluster was calculated, termed “A content.” Candidate PAS clusters were then grouped into three based on the presence of i) a canonical polyA signal (AATAAA or ATTAAA), ii) an alternative polyA signal (TATAAA, AGTAAA, AATACA, CATAAA, AATATA, GATAAA, AATGAA, AAGAAA, ACTAAA, AATAGA, AATAAT, AACAAA, ATTACA, ATTATA, AACAAG, AATAAG) or iii) no polyA signal in the 40 nucleotide window upstream of the PAS cluster. Different filtering thresholds were used for each group based on assessment of the nucleotide distribution profile for each group for deciles of “A content”: those with over 50% for canonical polyA signal clusters, over 40% for alternative polyA signal, and over 30% for no polyA signal, were removed. Finally PAS clusters within 200 nucleotides were merged, with the one with the most read counts kept as the indicative site. If two had the same count, the most 3′ cluster was kept. The clusters were assigned to genes, based on whether they overlapped annotated 3′ UTRs, or the 1 kb downstream of the annotated 3′ UTR, or an annotated gene in a hierarchical manner. Remaining intergenic PAS were discounted. This process generated our atlas of reliable PAS for quantification.

For quantification, all the sequencing reads were used (with any polyA tails trimmed using Cutadapt). These were then aligned against the human genome (GRCh38, with the Gencode V33 annotation) using STAR. For each experiment, BEDtools (Quinlan, 2014) was used to count the number of reads that mapped to a window 200 nucleotides upstream of the PAS in a strand-aware manner. This count table was used as the input for DRIMSeq (Nowicka and Robinson, 2016). Pairwise comparisons were done for each TDP-43 variant cell line between the knockdown and rescue condition. PAS that were either in genes with fewer than 10 reads in 75% of replicates across all conditions or themselves had fewer than 5 reads in 75% replicates in either knockdown or rescue conditions (whichever was lower) were filtered. DRIMSeq was used to fit the gene level Dirichlet-multinomial model and transcript level beta-binomial model to the data, using an additional covariate to account for the experimental batch. To assess for differential PAS usage, a likelihood ratio test was used, with Benjamini-Hochberg correction for multiple testing. A predefined threshold of p < 0.05 was deemed statistically significant. Any gene that had a significantly changing PAS in any pairwise comparison was considered to be a potentially regulated gene. We used the modeled proportion estimates as quantification of the usage of each PAS. Next, to derive those genes with robust regulation, we filtered out sites that had a less than 10% change in PAS usage (Table S4). For each gene, we identified one representative regulated PAS by selecting first the one that had the lowest adjusted p value and then breaking ties by selecting the one with the highest PAS usage.

To cluster genes with similar patterns of TDP-43 mutant rescue, we selected those genes for which the representative PAS usage change (dPAU) could be calculated for every mutant condition. We adjusted the direction of dPAU for each mutant rescue condition relative to the WT, such that it was always positive for the WT rescue. We clustered genes based on two measurements: i) the relative dPAU between WT and 316del346 and ii) the absolute dPAU between WT and 316del346, in order to consider both the direction and magnitude of change. We clustered the genes using partitioning by medoids (k-medoids clustering), as it is more robust to outliers and noise than k-means clustering, and used the average silhouette method to calculate the optimal number of groups into which to partition the genes.

To identify the partner PAS matching the representative PAS, we selected the partner site with the largest change in usage in the opposite direction to the representative site. This defined the two anchors of the 3′ UTR, between which we measured iCLIP binding signal and motif coverage.

### Quantification and statistical analysis

Information on statistics for image quantification is given in the ‘Statistics for image quantification’ chapter, statistical tests used is given in figure legends, and on statistics of analysis of differential pAsite in the chapter ‘Analysis of pAseq data’, and information of statistical tests used to determine significance of specific differences is described in figure legends.

## Data Availability

The sequencing data generated in this study have been deposited at EBI ArrayExpress under accession numbers “ArrayExpress: E-MTAB-9436” (iCLIP) and “ArrayExpress: E-MTAB-9410” (pAseq). All iCLIP data are available processed on the iMaps webserver: https://imaps.goodwright.org/collections/868/ All raw data have been deposited at Mendeley Data, https://doi.org/10.17632/834kstxzry.1. All code used to analyze the data and generate figures is available at https://github.com/ulelab/tdp43-mutants.

## Supplemental information

Table S1. List of iCLIP experiments, related to Figures 4, 5, 6, S4, S5, and S6

Table S2. iCLIP cDNA counts, related to Figure 5

Table S3. iCLIP cDNA counts, related to Figures 5 and S5

Table S4. PAS in each gene upon rescue by WT TDP-43 or each variant, related to Figure 7A
